# NF-κB-mediated EAAT3 upregulation in antioxidant defense and ferroptosis sensitivity in lung cancer

**DOI:** 10.1038/s41419-025-07453-y

**Published:** 2025-02-22

**Authors:** Donghua Wen, Wenjing Li, Xiang Song, Min Hu, Yueling Liao, Dongliang Xu, Jiong Deng, Wenzheng Guo

**Affiliations:** 1 Department of Laboratory Medicine, Shanghai East Hospital, Tongji University School of Medicine, Shanghai, 200120 China; 2 Breast Cancer Center, Shandong Cancer Hospital and Institute, Shandong First Medical University and Shandong Academy of Medical Sciences, Jinan, Shandong 250117 China; 3 College of Life and Environmental Science, Wenzhou University, Wenzhou, 325035 China; 4 Renji Hospital, Shanghai Jiao Tong University School of Medicine, Shanghai, 200025 China; 5 Medical Research Center, Affiliated Hospital of Binzhou Medical University, Binzhou, 256600 Shandong China

**Keywords:** Non-small-cell lung cancer, Stress signalling

## Abstract

Cellular glutathione (GSH) in lung cancer cells represents the most abundant antioxidant. GSH production is regulated not only by upregulated cystine/glutamate exchanger (xCT) but also by the involvement of glutamate transporters, specifically excitatory amino acid transporter 3 (EAAT3). Our prior research established that the uptake of glutamate via EAAT3 plays a pivotal role in driving cystine uptake through xCT, contributing to GSH biosynthesis during lung tumorigenesis. Nevertheless, the underlying mechanism governing the upregulation of EAAT3 remains enigmatic. In this study, we conducted a comprehensive reanalysis of publicly available data and employed the Gprc5a^–/–^/SR-IκB mouse model alongside in vitro cell experiments to elucidate the correlations between NF-κB and EAAT3 in lung cancer. We observed that EAAT3 knockdown, similar to NF-κB inhibition, led to the accumulation of reactive oxygen species (ROS) and increased sensitivity to ferroptosis induction by RAS-selective lethal 3 (RSL3). Mechanistic insights were obtained through chromatin immunoprecipitation and luciferase reporter assays, revealing that NF-κB induces EAAT3 expression via two putative cis-elements within its promoter. Furthermore, our investigation unveiled the upregulation of EAAT3 in a subset of clinical non-small cell lung cancer (NSCLC) tissues, exhibiting a positive correlation with the P65 protein. In addition, the inflammatory factor of smoking was found to augment EAAT3 expression in both human and murine experimental models. These findings collectively emphasize the pivotal role of the NF-κB/EAAT3 axis in managing antioxidant stress and influencing lung cancer development. Moreover, this research offers insights into the potential for a combined ferroptosis therapy strategy in lung cancer treatment.

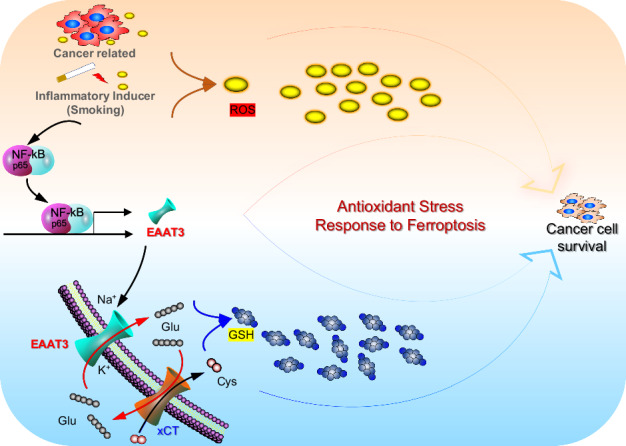

## Highlights

- NF-κB drives EAAT3 expression, key for GSH biosynthesis in lung cancer cells.
- EAAT3 knockdown increases ROS and enhances sensitivity to ferroptosis in lung cancer.
- EAAT3 upregulation in NSCLC tissues correlates with P65 protein levels.
- Smoking-induced inflammation boosts EAAT3 expression in lung cancer models.
- Potential for combined ferroptosis therapy targeting NF-κB/EAAT3 axis in lung cancer.

## Introduction

Lung cancer continues to be one of the most widespread and lethal malignancies globally, with smoking constituting a significant risk factor for its onset [1]. In recent years, extensive research has unveiled the complex interplay between chronic inflammation and redox imbalance in the context of lung carcinogenesis [2–4]. Chronic exposure to tobacco smoke triggers two significant responses. Firstly, it stimulates lung epithelial cells. This persistent exposure to various inflammatory stimuli, including environmental toxins, infections, and autoimmunity, creates an inflammatory microenvironment that enhances oncogenic processes and facilitates tumor initiation [5–7]. Within this inflammatory milieu, a complex network of pro-inflammatory cytokines, chemokines, and immune cells orchestrates activities that promote tumor cell proliferation, angiogenesis, and immune evasion [8–10]. Furthermore, chronic inflammation can lead to DNA damage and genomic instability, further elevating the risk of malignant transformation [4].

A second significant response to chronic tobacco smoke exposure is the generation of reactive oxygen species (ROS), which overwhelm the cellular antioxidant defense system, primarily centered around glutathione (GSH) [11, 12]. This persistent oxidative stress leads to an ongoing inflammatory response and subsequent cellular damage, fostering the initiation and progression of lung cancer [13, 14]. One area where the effects of ROS have gained significant attention is in the development of lung cancer. A comprehensive understanding of the intricate interplay between ROS and antioxidant defense systems, such as GSH, is crucial for elucidating disease progression and developing potential therapeutic strategies in lung cancer. ROS-induced DNA damage, genomic instability, and altered signaling pathways have all been implicated in the initiation and promotion of lung carcinogenesis [15–17]. In contrast, GSH, a vital intracellular antioxidant, plays a pivotal role in maintaining redox homeostasis by scavenging ROS and safeguarding cells against oxidative damage [18]. The dysregulation of ROS and GSH balance can tip the scales toward an oxidatively stressed microenvironment, contributing to the transformation and survival of cancerous cells.

This work builds on our previous research, which illustrated that active glutamate uptake through solute carrier family 1 member 1 (EAAT3/SLC1A1) plays a pivotal role in driving cystine uptake via X_c_^–^ for GSH biosynthesis in lung tumorigenesis [19]. Cellular GSH in cancer cells is not only determined by upregulated X_c_^–^ but also by dysregulated glutamate transporters [19]. EAAT3 serves as the predominant amino acid transporter for glutamate and aspartate, making it particularly relevant to cancer development, especially within the realm of cancer metabolism [20, 21]. While the dysregulation of EAAT3 has been associated with metabolic reprogramming and the progression of various solid tumors, the precise regulatory mechanisms governing EAAT3 remain insufficiently understood. Drawing on our previous mouse model, Gprc5a-knockout (KO) mice not only developed spontaneous lung tumors [22], but also exhibited an enhanced inflammatory response within lung tissues [23–25]. Recent research has proposed that the NF-κB repressing factor (NKRF) specifically binds to the EAAT3 promoter, resulting in transcriptional downregulation of EAAT3 expression [26]. When considering our RNA sequencing data, we hypothesize that inflammation may play a role in modulating the expression of EAAT3.

In this study, our objective is to demonstrate the significant role of the NF-κB pathway in the anti-ROS system. We aim to show that the inhibition of NF-κB can induce ROS production by down-regulating the expression of EAAT3. Utilizing the Gprc5a^–/–^/SPC-SR-IκBα mouse model, we have discovered that NF-κB induces the expression of EAAT3 through two potential cis-elements in its promoter. Furthermore, in clinical cases involving smokers, we have verified the correlation between EAAT3 and NF-κB in the context of persistent chronic inflammation caused by smoking.

## Materials and methods

### Reagents and antibodies

#### Reagents

QNZ-EVP4593(S4902), BMS-345541 (S8044), BSO (S2433), doxycycline (S5159), puromycin 2HCL (S7417), cocktail (B14002), 2x SYBR Green qPCR master mix (B21203), NNK (E0651) were from Selleck.cn (Houston, TX, USA). GSH-MEE (G1404), hydrogen peroxide solution (H_2_O_2_, 95321), TNFα (SRP3177), and DAPI (D9542) were from Sigma-Aldrich (St. Louis, MO, USA). Dual-Luciferase® Reporter Assay System (E1910) was from Promega (Madison, WI, USA). CellROX™ Orange Reagent (C10443) was from Thermo Fisher Scientific (Waltham, MA, USA). RIPA buffer (9806) was from Cell Signaling Technology (Danvers, MA, USA). Immobilon Western Reagents (15015B4) was from Millipore (Burlington, MA, USA). siRNA-P65(sc-29410) and siRNA-EAAT3(sc-41940) were from Santa Cruz Bio (Dallas, TX, USA). ^13^C_6_-^15^N_2_-labeled cystine (CNLM-4244-H-PK) was from Cambridge Isotope Laboratories (Tewksbury, MA, USA).

#### Antibodies

Anti-EAAT3 antibody (12686-1-AP) for IHC was from Proteintech (Rosemont, IL, USA). Anti-EAAT3 antibody (14501), anti-BAX (5023), anti-p-P65 (3033), anti-P65 (8242), anti-H3K27ac (8173), anti-V5-tag (13202) were from Cell Signaling Technology (Danvers, MA, USA). Anti-RNA polymerase II (ab5131) was from Abcam (Cambridge, UK). Anti-RAI3 (Gprc5a; sc-98884) was from Santa Cruz Bio (Dallas, TX, USA). Anti-β-Actin-HRP (PM053-7), anti-DDDDK-tag (M185-3L) were from MBL (Nagoya, Aichi, Japan). Anti-FLAG® M2 Affinity Gel (A2220) was from Sigma-Aldrich (St. Louis, MO, USA). The antibodies were diluted according to manufacturers’ instructions.

### Cell lines and cell culture

Mouse tracheal epithelial cells (MTECs) were obtained from normal tracheal tissue of 3-week-old Gprc5a^+/+^ and Gprc5a^–/–^ mice (C57BL/6 X129sv) as described [27, 28]. The MTECs were cultured in K-SFM supplemented with epithelial growth factor (EGF, 5 ng/ml) and bovine pituitary extract (50 μg/ml, Invitrogen; Carlsbad, CA, USA). Human embryonic kidney cells HEK293T, normal lung epithelial cells (16HBE, HBEC), and NSCLC cells (A549, PC9) were obtained from the American Type Culture Collection (ATCC; Manassas, VA, USA) and were tested and authenticated by DNA typing at Shanghai Jiao Tong University Analysis Core (Shanghai, China). The mouse lung adenocarcinoma cell line 1601 was isolated from lung tumor growth in Gprc5a^–/–^ mice. The HBEC cells were cultured in K-SFM, and the other cells were cultured in DMEM essential medium with 10% fetal calf serum at 37 °C in a humidified incubator in an atmosphere of 95% air and 5% CO_2_.

### Immunoprecipitation and western blotting

Cells were lysed with RIPA buffer, and 500 μg whole cell lysate was precipitated with 2 μg agarose-conjugated antibody against Flag (Flag beads) and normal IgG (as a negative control). PBS with 0.05% Tween-20 was used to wash the beads five times. Then, an equal amount of protein loading buffer was added and boiled 5 min. All samples were separated by SDS-PAGE and transferred to NC membranes (GE Healthcare Life Science, Boston, MA, USA; Lot.G9597136). The 5% (w/v) no-fat dry milk in TBST was used to block non-specific binding sites. The blocked membranes were incubated with the specific primary antibodies diluted in 5% BSA (Sigma-Aldrich) with 0.05% sodium azide overnight on a shaking bed at 4 °C. HRP-conjugated secondary antibody was incubated at room temperature for 1 h. Finally, the membranes were visualized by exposure to Immobilon Western reagents.

### CellROX (FACS)

NSCLC cells were plated in 6-well plates. The cells were transfected with 5 µM siRNA-P65 for 24 h at 37 °C and treated with GSH-MEE 2 mM for 12 h. The cells were then stained with 5 µM of CellROX® Orange reagent by adding the probe to the complete medium and incubating the cells at 37 °C for 30 min. The cells were then washed with PBS and analyzed on FACS. Finally, the data were analyzed in the FlowJo 7.6 software.

### CellROX (IF)

NSCLC cells were plated in six-well plates with cover slip. The cells were transfected with 5 µM si-RNA-P65 for 24 h at 37 °C and treated with GSH-MEE 2 mM for 12 h. The cells were then stained with 5 µM of CellROX® Orange reagent by adding the probe to the complete medium and incubating the cells at 37 °C for 30 min. The cells were then washed with PBS and put on a glass slide. A Nikon confocal microscope was used for the analysis and imaging.

### Transfection and co-immunoprecipitation

A549 and PC9 cells were transfected with siRNA-P65 or siRNA-EAAT3 through Lipo 2000 (siRNA: Lipo 1:1). HEK293T cells were transfected with Flag-EAAT3, V5-SLC7A11, and pcDNA3.1-Vector through PEI (plasmid : PEI 1:4) transfection reagents, which replaced the medium after 4–6 h. After 24 h, we removed the culture media and washed cells with ice-cold PBS. Cells were centrifuged at 400×*g* for 10 min at 4 °C. After the second wash, we removed the supernatant completely and resuspended the cell pellets in 1 mL of ice-cold RIPA lysis buffer containing protease or phosphatase inhibitors. We then placed the tube on ice for 30 min, with occasional mixing. Next, we centrifuged the cell lysate at 10,000×*g* for 15–30 min at 4 °C, then added 50 ul Flag-M2-beads and incubated overnight on a rotator at 4 °C. Subsequently, we washed with cold PBS three times and lysed the beads. Finally, we performed a western blot analysis.

### Quantitative real-time PCR

Cells and tissues were first lysed using Trizol, then the total RNA was extracted with RNA Easy Fast Tissue/Cell Kit (Tiangen, city, ST, Ctry; 4992732) and cDNA was prepared from 1.5 μg of the total. All mRNA was detected by an ABI 7300 real-time PCR machine using the 2x SYBR Green qPCR Master Mix. Primers used listed in Table 1.

**Table 1:** Used qPCR primers and ChIP-qPCR primers.

**qPCR_Primers**	
Gprc5a-F	GACACACTCTATGCACCTTATTC
Gprc5a-R	ACAGACCTTGTCTACTCCAG
β-Actin-F	GCTCTTTTCCAGCCTTCCTT
β-Actin-R	CTTCTGCATCCTGTCAGCAA
EAAT3-F	GCGAGGAAAGGATGCGAGT
EAAT3-R	GCTGTGTTCTCGAACCAAGACT
Eaat3-F	GCCGCTTCCTACGGAATCAC
Eaat3-R	GTCCTCGAACCACGACTCCTA
RELA-F	ATGTGGAGATCATTGAGCAGC
RELA-R	CCTGGTCCTGTGTAGCCATT
RelA-F	TGCGATTCCGCTATAAATGCG
RelA-R	ACAAGTTCATGTGGATGAGGC
BAX-F	CCCGAGAGGTCTTTTTCCGAG
BAX-R	CCAGCCCATGATGGTTCTGAT
SLC1A2-F	CCTGACGGTGTTTGGTGTCAT
SLC1A2-R	CAAGCGGCCACTAGCCTTAG
SLC1A3-F	AGCAGGGAGTCCGTAAACG
SLC1A3-R	AGCATTCCGAAACAGGTAACTTT
GRM1-F	CCAGCGATCTTTTTGGAGGTG
GRM1-R	TGGTGATGGACTGAGAAGAGG
GRM5-F	ATGCCGGGTGACATCATTATTG
GRM5-R	TGAATGCCATACTGTTCACGG
GRM8-F	CGAGGGAAAGCGATCAGCC
GRM8-R	CCCATCCACCCGTATGGAA
**ChIP-qPCR_Primers**	
EAAT3-p1-F	AAGGGCAGCAAGAAGGCC
EAAT3-p1-R	TGGGCGAGAAGAAAGCTCAA
EAAT3-p2-F	CCTCTCAGGAGAAGCTGAA
EAAT3-p2-R	CGGTGGTGTTGAAGAGCAGCAA
IL8-F	TGGGCCATCAGTTGCAAA
IL8-R	AC TTATGCACCCTCATCTTTTCATT

### Animals

Gprc5a^–/–^ mice were generated in a mixed background of 129sv x C57BL/6 as described previously [22, 29]. Crossbreeding *Gprc5a*-ko mice with SPC-dnIκB-α mice to generated *Gprc5a*^–/–^/SPC-dnIκB-α mice, in which dominant negative (dn) or super-repressor (SR) IκBα gene is driven by the type II cell marker surfactant protein C (SPC) promoter [30]. Eight-week-old Gprc5a^–/–^, Gprc5a^+/+^, and *Gprc5a*^–/–^/SPC-dnIκB-α mice were injected with NNK (NNK,100 mg/kg body weight, i.p. dissolved in 0.9% NaCl) for 2 weeks. Ten months later, all mice were sacrificed for H&E staining analysis, followed by extraction of protein and RNA for metabolic profiling analysis.

Eight-week-old C57BL/6 mice were injected with 1601 (5 × 10^5^ cells). BSO (1.5 mg/kg) was orally gavaged every day for 10 days from the day’s injected cells. Two weeks later, tumor volume was measured, and further analysis was conducted.

### Construction of plasmids

pcDNA3.1( + )-EAAT3-Flag and pcDNA3.1( + )-SLC7A11-V5 plasmids were constructed as described previously [31]. The mutated luciferase plasmid construction is based on the traditional plasmid construction protocol. Briefly, we designed primers with desired mutations in the target gene of the luciferase reporter plasmid (pGL3). Then, we conducted PCR amplification and purification, followed by digestion of the purified PCR product and the original luciferase reporter plasmid with appropriate restriction enzymes. Finally, we performed ligation and transformation, then selection and sequencing.

### IHC and scores

A tissue microarray was stained to identify EAAT3, GPRC5A, P65, and BAX proteins. The mouse lung tissues were stained to identify Eaat3. The IHC protocol and score method was performed as previously described [32].

### Luciferase assay

HEK293T cells were cultured in 24-well plates and transfected with EAAT3 promoter-driving luciferase plasmid and its mutations (MutA, MutB, and MutAB). After 24 h, cells were lysed in lysis buffer per the instructions of the Dual-Luciferase® Reporter Assay System. The luminometer reader was used to detect the luciferase activity.

### ChIP

A549 cells were cultured in a 15 cm plate at a density of about 90% and treated with or without Dox. We performed cross-linking of chromatin and associated proteins using 1% formaldehyde, followed by quenching with glycine. Next, cell samples were lysed in a lysis buffer (1% SDS, 10 mM EDTA, 50 mM Tris, and protease inhibitor mixture, pH 8.0). The chromatin was then sheared into fragments of desired size using enzymatic digestion. To reduce non-specific binding, pre-clearing was performed by incubating with protein G beads coupled with a non-specific antibody. The chromatin was subsequently immunoprecipitated with specific antibodies (RNA poly II and H3K27ac were positive control, NIgG was the negative control) against the target protein and incubated overnight at 4 °C with gentle rotation. After capturing the antibody-bound chromatin complexes with 60 ul protein G beads, a series of wash steps were carried out to remove non-specifically bound proteins and DNA. The immunoprecipitated chromatin was eluted from the beads and subjected to decrosslinking at 65 °C. Proteinase K treatment was then performed to remove proteins, followed by DNA purification. The purified DNA was resuspended in TE buffer for PCR analysis (10 mM Tris-HCl and 1 mM EDTA, pH 8.0). Enriched DNA was analyzed using quantitative PCR (qPCR) with primers specific to the target genomic regions to identify genome-wide binding sites of the target protein (Primers used are listed in Table 1). Data analysis involved normalization to appropriate controls and determining the enrichment of the target protein.

### CSE preparation

The aqueous cigarette smoke extract (CSE) was prepared with slight modifications to a previously established method [33]. In short, 10 mL of serum-free sterile DMEM medium was placed into a 60 mL plastic syringe. Then, 40 mL of cigarette smoke from one puff of a 3R4F filtered cigarette (from the Kentucky Tobacco Research and Development Center, University of Kentucky, Lexington, KY, USA) was drawn into the syringe. The contents were mixed by vigorous shaking for 30 s. One cigarette was used for every 10 mL of the medium, and each cigarette provided 11 puffs. The resulting CSE solution, filtered through a sterile 0.22-μm filter, was considered the 100% stock solution. The CSE was then diluted with culture medium before use.

### GSH colorimetric detection

Cell lysate: Washed cell pellets are resuspended at 1–10 × 10^6^ cells/mL in cold 5% SSA and are lysed and deproteinized by vigorous vortexing. Incubate cells at 4 °C for 10 min followed by centrifugation for 10 min at 14,000 rpm and 4 °C. Tumor tissue: Fresh tumor tissue is washed with ice-cold PBS to remove blood then blotted on filter paper before recording wet weight.

The GSH assay was performed following the protocol provided by the GSH Colorimetric Detection Kit (ARBOR ASSAYS, #K006-H1, MI, USA). Briefly, samples were homogenized at a ratio of 10 mg tissue per 250 µL of ice-cold 5% SSA (sulfosalicylic acid). The homogenate was incubated for 10 min at 4 °C, followed by centrifugation at 14,000 rpm for 10 min at 4 °C to remove precipitated proteins. The supernatant was then collected and diluted 1:5 with the assay buffer by mixing 1 part supernatant with 4 parts assay buffer, resulting in a final SSA concentration of 1%.

### Cystine uptake assay

For in vivo detection of tumor cell cystine uptake, ^13^C_6_-^15^N_2_-labeled cystine was administered to mice via intraperitoneal (i.p.) injection. The mice were treated daily with BSO following the approved animal experiment protocol. The labeled cystine was injected 6 h prior to tumor excision. Fresh tumor tissues were then prepared for UPLC/MS analysis, following the previously described method in our study [19].

### Transmission electron microscopy (TEM)

PC9 cells were cultured under standard conditions, harvested at 70–80% confluence, and fixed in 2.5% glutaraldehyde in 0.1 M sodium cacodylate buffer at 4 °C for 2 h. After washing with buffer, samples were post-fixed in 1% osmium tetroxide, dehydrated through a graded ethanol series, and embedded in epoxy resin. Ultrathin sections (70–90 nm) were prepared using an ultramicrotome, stained with 2% uranyl acetate and Reynold’s lead citrate, and imaged using a JEOL JEM-1400 transmission electron microscope operated at 80 kV.

### Public dataset and bioinformatics

The GEO datasets were downloaded from NCBI (www.ncbi.nlm.nih.gov). The datasets (GSE17737 and GSE64614) were used for analysis of the inflammation-inducing factor (smoking), which increased the expression of EAAT3. The dataset (GSE44619) was used for analysis of the correlation between NF-κB and EAAT3.

Fastp software (v0.20.0) was used to trim the adapter and remove low-quality reads to get high-quality clean reads. Clean reads were aligned to the human hg38 reference genome using bowtie2 software (v2.2.4). MACS2 software (v2.2.7.1) was used for peak calling. ChIPSeeker R package (v1.30.3) was used for peak annotation. Homer software (v4.11) was used to identify motifs. The enriched peaks were visualized in IGV (v2.4.10) software. GO and KEGG pathway analysis were performed based on the promoter-enriched peaks (or promoter-enriched differentially enriched regions) associated genes using the ClusterProfiler R package (v3.18.1).

The EAAT3 and RELA expression heatmaps were from https://xenabrowser.net/. The correlation of EAAT3 and CXCL1, BCL2, RELA were from http://timer.cistrome.org/ [34]. The transcription factor binding sites were predicted in The Contra v3 (http://bioit2.irc.ugent.be/contra/v3) [35], and then we re-valued the binding sites using JASPAR (https://jaspar.genereg.net/) [36]. The tHPA website (https://www.proteinatlas.org/) was used for the analysis of the protein expression in human tumor tissues.

### Statistical analyses

Data were analyzed using SPSS Statistics software (IBM, Version 19) and presented as the mean ± standard deviation. The differences of results were compared using a two-tailed paired *t*-test assuming unequal distribution. Multiple group comparisons used one-way analysis of variance (ANOVA); a *p*-value < 0.05 was considered statistically significant.

## Results

### NF-κB was involved in the expression of EAAT3

**Fig. 1:**
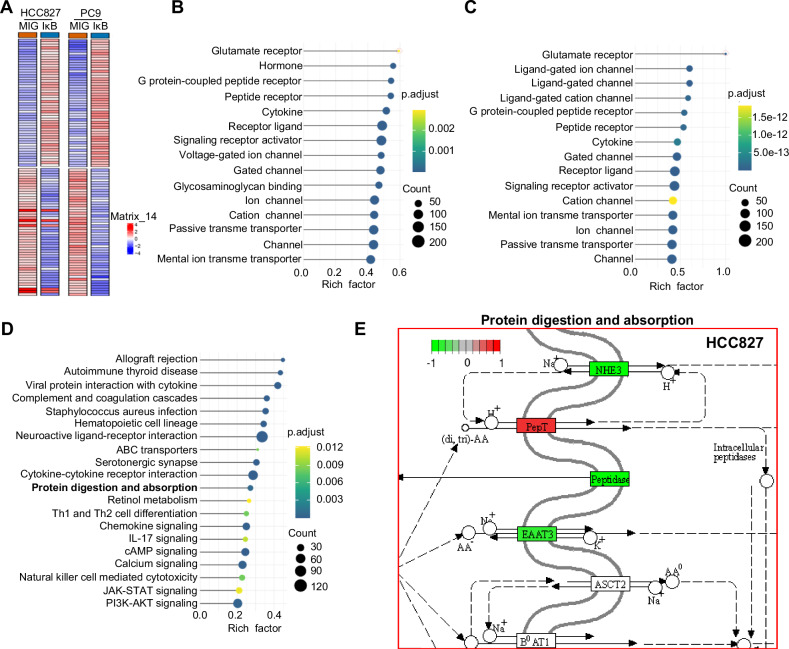
NF-κB was involved in the expression of EAAT3. **A** Heatmap of different gene expression in two cell lines from GSE44619. IkB: Cells were transduced with MIG retrovirus expressing IκBɑ-SR mutant (S32, S36 to A mutations); MIG: Cells were transduced with control retrovirus. **B** GO enrichment analysis of the differentially expressed downregulated genes in HCC827. **C** GO enrichment analysis of the differentially expressed downregulated genes in PC9. **D** KEGG analysis on common differential genes of both cell lines. **E** EAAT3 was enriched in the protein digestion and absorption pathway in HCC827 cells.

In our previous study, we reported that GPRC5A deficiency leads to the induction of EAAT3, facilitating glutamate recycling and GSH biosynthesis. However, the underlying mechanism remained unclear. Notably, the *Gprc5a*-ko mouse lung cancer model exhibits two significant characteristics: the spontaneous development of lung tumors and a persistent state of inflammation. Extensive data indicate that NF-κB activity is significantly elevated in the lungs of *Gprc5a*-ko mice and MTEC-KO cells (mouse lung epithelial cells lacking Gprc5a) when compared to wild-type (WT) counterparts [28, 37]. To shed light on the regulatory mechanism of EAAT3 in lung cancer, we postulated a plausible hypothesis implicating the NF-κB signaling pathway in EAAT3 regulation within lung cancer. In an initial endeavor to substantiate this hypothesis, we conducted a reanalysis of publicly accessible data from the GSE44619 dataset within the GEO database [38]. This dataset offers insights into the impact of NF-κB inhibition on gene expression in human lung cancer cell lines. For our analysis, we selected two human lung adenocarcinoma cell lines, HCC827 and PC9. We performed a comparative analysis of differentially expressed genes between the group treated with IκBɑ super-repressor (SR) mutant and the control group (Fig. 1A). Subsequently, we conducted GO enrichment analysis on the genes that exhibited downregulation, revealing a statistically significant enrichment of the glutamate receptor pathway in both ADC cell lines (Fig. 1B, C). To gain a deeper understanding of this correlation, we carried out KEGG analysis on the common differentially expressed genes in both cell lines (Fig. 1D). Among these differential signaling pathways, EAAT3 was enriched within the protein digestion and absorption pathway. Notably, when the lung cell lines were infected with a retrovirus expressing the IκBɑ-SR mutant, EAAT3 expression exhibited a decrease (Fig. 1E and Supplementary Fig. 1A, B). We also investigated the connection between NF-κB and EAAT3 in the KP model (Kras^G12D/+^; Trp53^fl/fl^) using the publicly available GEO dataset (GSE206644) [39], which provides comparative RNA-seq gene expression profiling data for KP tumors. Our analysis revealed a positive correlation between the NF-κB pathway and EAAT3 expression in the KP model (Supplementary Fig. 1C). These results collectively highlight the involvement of the NF-κB signaling pathway in the regulation of EAAT3 expression.

### NF-κB induced the expression of EAAT3 in vitro and in vivo

**Fig. 2:**
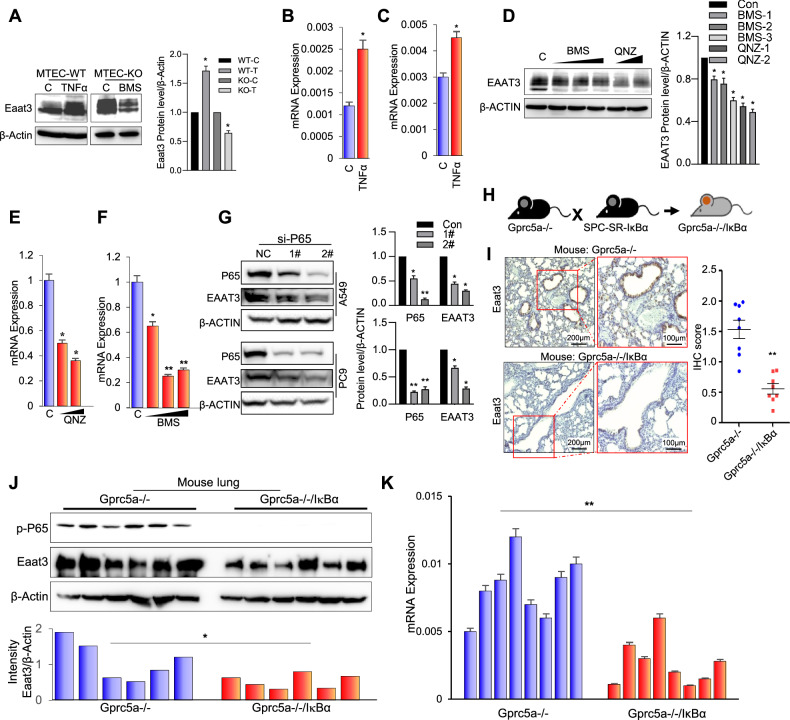
NF-κB induced the expression of EAAT3 in vitro and in vivo. **A** Eaat3 expression in mouse lung normal epithelial cell MTEC and MTEC-KO-Gprc5a cells treated with TNFα (10 ng/mL) and BMS 345541 (10 μM). **B** EAAT3 mRNA expression in human normal lung 16HBE epithelial cells treated with TNFα (10 ng/mL). **C** EAAT3 mRNA expression in human normal lung HBEC epithelial cells treated with TNFα (10 ng/mL). **D** EAAT3 expression in lung cancer A549 cell line treated with NF-κB inhibitor BMS 345541 (5, 10, and 15 μM) and QNZ-EVP4593(5, 10 μM). **E**,**F** EAAT3 mRNA expression in A549 cells treated with BMS (5, 10, and 15 μM) and QNZ-EVP4593(5 and 10 μM). **G** P65 and EAAT3 expression in A549 and PC9 cells transfected with siRNA-P65. **H**,**I** The expression of Eaat3 in Gprc5a−/− and Gprc5a−/−/SPC-dnIκBα mouse lung tissues by IHC (x10). **J** The expression of Eaat3 in Gprc5a−/− and Gprc5a−/−/SPC-dnIκBα mouse lung tissues by western blot (*n* = 6). **K** The expression of Eaat3 in Gprc5a−/− and Gprc5a−/−/SPC-dnIκBα mouse lung tissues by real-time PCR (*n* = 8). *p < 0.05, **p < 0.01, error bars represent SEM.

To investigate the involvement of the NF-κB signaling pathway in the upregulation of EAAT3, we assessed EAAT3 expression in MTEC-KO and MTEC-WT cell lines. Notably, immunoblot analysis revealed that TNFα induced EAAT3 expression in MTEC-WT cells, while the NF-κB inhibitor BMS 345541 suppressed EAAT3 expression in MTEC-KO cells (Fig. 2A). Additionally, qPCR analysis demonstrated that TNFα induced EAAT3 expression in human lung epithelial cell lines, HBEC and 16HBE (Fig. 2B, C). Given the presence of several members in the glutamate transporter family, we also examined the expression of key glutamate transporters in human epithelial cell lines following TNFα treatment. In 16HBE cells, the expression of SLC1A2, SLC1A3, GRM1, GRM5, and GRM8 increased with TNFα treatment (Supplemental Fig. 2A–E). However, in HBEC cells, only GRM1 was induced by TNFα treatment (Supplemental Fig. 2F–J). Furthermore, the expression of EAAT3 in the NSCLC cell line A549 was dose-dependently repressed by the NF-κB inhibitors BMS 345541 and QNZ-EVP4593 at both the protein and mRNA levels (Fig. 2D–F). Additionally, SLC1A2 and GRM8 were found to be inhibited by BMS, while several other members, such as SLC1A3, GRM1, and GRM5, exhibited the opposite effect (Supplemental Fig. 2K–O). Moreover, after silencing the expression of P65 with siRNA, EAAT3 expression decreased (Fig. 2G). In summary, these observations collectively support the notion that EAAT3 expression is induced by NF-κB signaling.

To assess whether the NF-κB pathway plays a crucial role in EAAT3 expression in an in vivo setting, we generated Gprc5a-ko/SPC-dnIκB-α mice by crossbreeding *Gprc5a*-ko mice with SPC-dnIκB-α mice. In the latter, a dominant negative (dn) or super-repressor (SR) IκBα gene is driven by the type II cell marker surfactant protein C (SPC) promoter (Fig. 2H) [30]. In a prior study, we demonstrated that TNFα-induced NF-κB activity was significantly suppressed in the lungs of Gprc5a-ko/SPC-dnIκB-α mice when compared to *Gprc5a*-ko mice [37]. Notably, in situ IHC staining revealed that Eaat3 expression was notably diminished in the tissues from Gprc5a-ko/SPC-dnIκB-α mice compared to those from *Gprc5a*-ko mice (Fig. 2I). Immunoblot analysis further substantiated these findings by showing a clear reduction in Eaat3 expression in lung tissues of Gprc5a-ko/SPC-dnIκB-α mice in contrast to those of *Gprc5a*-ko mice (Fig. 2J). Similarly, qPCR analysis demonstrated that the downregulation of Eaat3 occurred at the mRNA level (Fig. 2K). Consequently, these results confirm the essential role of NF-κB signaling in Eaat3 expression within lung epithelial cells in vivo.

### Inhibition of NF-κB increased ROS accumulation

**Fig. 3:**
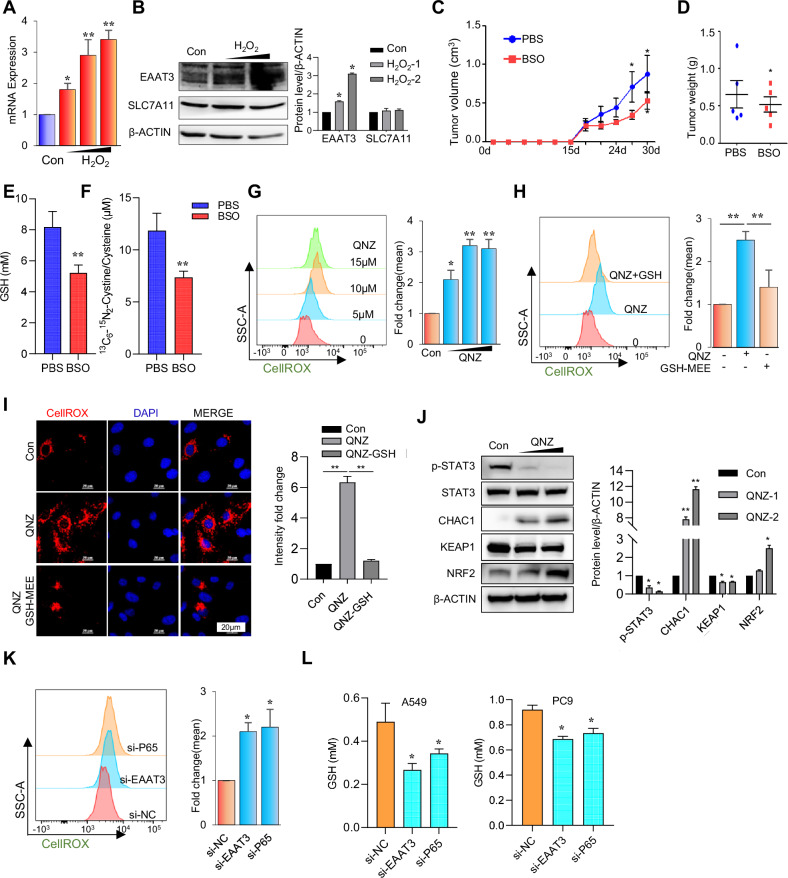
Inhibition of NF-κB increased the accumulation of ROS. **A** The mRNA expression of EAAT3 in 16HBE cells treated with H_2_O_2_ (50, 100, and 200 μM) for 24 h. **B** The protein expression of EAAT3 and SLC7A11 in 16HBE cells treated with H_2_O_2_ (50 and 100 μM). **C**,**D** The tumor volume and weight in C57BL/6 injected with mouse lung cancer cell line 1601, then treated with BSO (1601 cells : 1 × 10^6^, *n* = 5). **E** The GSH Colorimetric Kit detects the decreased GSH levels in tumor tissues following treatment with BSO. **F** UPLC/MS showed the concentration of labeled cystine and cysteine in tumor tissues. **G** FACS shows the ROS level in A549 cells treated with QNZ (5, 10, and 15 μM). **H** FACS showing the ROS level in A549 cells treated with QNZ (10 μM) and GSH-MEE (2 mM). **I** IF showing the ROS level in A549 cells treated with QNZ (10 μM) and GSH-MEE (2 mM) (scale bar: 20 µm). **J** The protein expression of ROS markers CHAC1, NRF2, and KEAP1 in A549 cells treated with QNZ (5 and 10 μM). **K** FACS showing the ROS level in A549 cells silencing EAAT3 and P65 with siRNA. **L** GSH colorimetric detects the GSH concentration in A549 and PC9 cells silencing EAAT3 and P65. *p < 0.05, **p < 0.01, error bars represent SEM.

As demonstrated previously, EAAT3 plays a pivotal role in enhancing glutamate uptake for GSH synthesis, contributing to the aggressive characteristics of lung cancer cells. Consequently, we hypothesize that EAAT3 may function as a sensor and responder to ROS. Exposure to hydrogen peroxide (H_2_O_2_) led to a dose-dependent induction of EAAT3 expression at both the mRNA and protein levels (Fig. 3A, B), while no significant change was observed in the xCT antiporter (Fig. 3B). In the context of tumor growth, substantial ROS are generated, which can be detrimental to the tumor and necessitate the synthesis of GSH for neutralization. We confirmed this phenomenon through an experiment involving the subcutaneous transplantation of C57BL/6 mice with 1601 tumor cells. Treatment with the GSH inhibitor BSO significantly suppressed tumor growth (Fig. 3C, D). To confirm the tumor-suppressive effect of BSO treatment, we utilized a GSH colorimetric assay to demonstrate a significant reduction in GSH levels following BSO exposure (Fig. 3E). Additionally, we employed ^13^C_6_-^15^N_2_-labeled cystine to evaluate cystine uptake in tumor cells post-BSO treatment. Given that cystine is rapidly reduced to cysteine intracellularly, we measured the combined levels of labeled cystine and cysteine to comprehensively estimate cystine uptake. UPLC-MS analysis revealed a decrease in the levels of labeled cystine and cysteine after BSO treatment (Fig. 3F), indicating reduced cystine uptake. In cancer cells, cellular GSH levels are influenced not only by upregulated xCT (SLC7A11) but also by altered glutamate transporters, particularly EAAT3. The upregulated glutamate transporter EAAT3 drives cystine uptake via xCT, facilitating GSH synthesis in lung cancer cells. In light of these findings, it is reasonable to speculate that EAAT3 and xCT might interact at the cell surface. To validate this hypothesis, we conducted a co-immunoprecipitation (co-IP) experiment. However, the results indicated that these two molecules do not interact with each other (Supplemental Fig. 3).

As NF-κB signaling plays a role in EAAT3 expression, we posited that inhibiting NF-κB could result in the accumulation of ROS. To investigate this hypothesis, we utilized QNZ to inhibit P65 expression in the A549 and PC9 cell lines. Flow cytometry assays revealed a dose-dependent increase in ROS levels with QNZ treatment (Fig. 3G and Supplementary Fig. 4A). Furthermore, we employed GSH-MEE to neutralize ROS, which demonstrated a reduction in ROS levels after GSH-MEE treatment in cells previously exposed to QNZ (Fig. 3H and Supplementary Fig. 4B). Immunofluorescence analysis confirmed the elevated ROS levels in cells with an inhibited NF-κB pathway (Fig. 3I and Supplementary Fig. 4C). Notably, the ROS response markers CHAC1 and NRF2 also increased in cells treated with QNZ. Furthermore, the inhibitory molecules of NRF2, KEAP1, showed reduced levels (Fig. 3J). Subsequently, we utilized siRNA to silence P65 and EAAT3 expression in the A549 and PC9 cell lines. As demonstrated, silencing either EAAT3 or P65 led to the accumulation of ROS (Fig. 3K and Supplementary Fig. 4D) and a reduction in GSH levels (Fig. 3L), indicating that NF-κB-mediated induction of EAAT3 plays a key role in regulating GSH synthesis in tumor cells. In summary, these findings collectively suggest that inhibition of NF-κB activation results in increased ROS production, likely mediated through the involvement of EAAT3.

### Inhibition of NF-κB/EAAT3 increased ferroptosis sensitivity

**Fig. 4:**
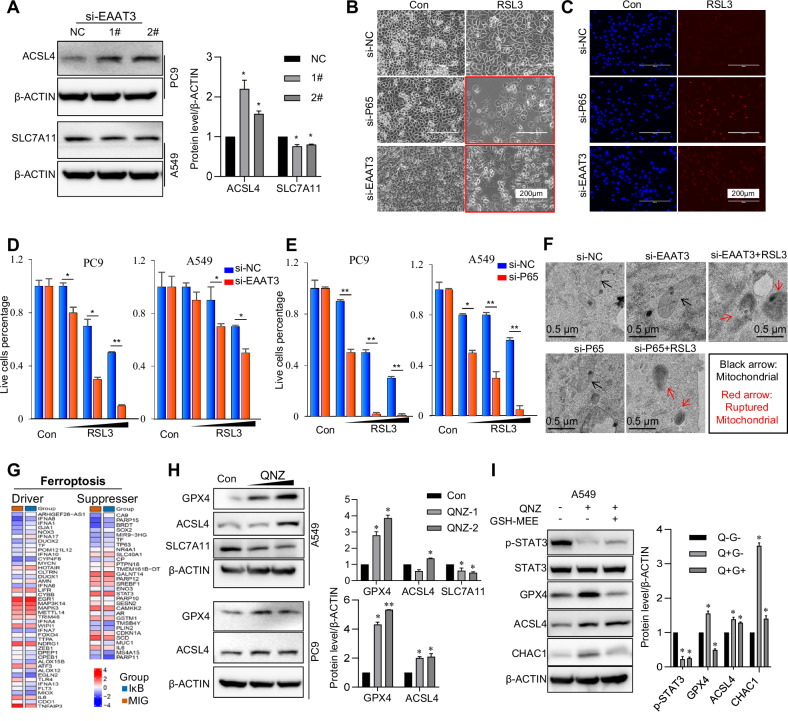
Inhibition of NF-κB/EAAT3 increased ferroptosis sensitivity. **A** The expression of ferroptosis marker ACSL4 and SLC7A11 in PC9 and A549 cells, respectively. **B** RSL3 (5 μM) treatment induced more cell death in silenced P65 or silenced EAAT3 in PC9 cells. **C** PI staining in PC9-siRNA-P65 and PC9-siRNA-EAAT3 cells with RSL3 treatment. **D** The live cell number count in siRNA-EAAT3 cells with dose-dependent RSL3 (5, 10, and 20 μM). **E** The live cell number count in siRNA-P65 cells with dose-dependent RSL3 (5, 10, and 20 μM). **F** TEM shows the morphology of mitochondria in si-EAAT3 and si-P65 PC9 cells with or without RSL3 treatment (RSL3: 5 μM). **G** Different ferroptosis driver genes and suppresser genes expression in PC9 cell from GSE44619. **H** The protein expression of ferroptosis marker GPX4, ACSL4, and SLC7A11 in cells with QNZ (5 and 10 μM) treatment. **I** The protein expression of ferroptosis marker GPX4 and ACSL4, the ROS marker CHAC1 in cells with QNZ (10 μM) and GSH-MEE (2 mM) treatment. *p < 0.05, **p < 0.01, error bars represent SEM.

As is widely recognized, the generation of ROS plays a central role in initiating lipid peroxidation, particularly the oxidation of polyunsaturated fatty acids within cellular membranes. ROS-mediated oxidative stress can lead to ferroptosis. Therefore, we assessed ferroptosis markers in silenced cell lines. Upon silencing EAAT3, ACSL4 levels increased in PC9 cells, while SLC7A11 levels decreased in A549 cells, suggesting that these cells may become more sensitive to ferroptosis (Fig. 4A). Similarly, cells with silenced P65 exhibited increased sensitivity to ferroptosis induced by RSL3, with a similar trend observed in EAAT3-silenced cells (Fig. 4B and Supplementary Fig. 5A). It is worth noting that while cell death also occurred in control cells, siRNA-treated cells experienced a higher rate of cell death compared to the control cells, with a statistically significant difference. Nevertheless, both groups shared a common outcome, with the majority of cells succumbing to death. To validate this cell death, PI staining revealed more cell death in si-P65 and si-EAAT3 cells following RSL3 treatment (Fig. 4C). Furthermore, when we counted the number of live cells in si-P65 and si-EAAT3 cells exposed to RSL3 treatment, both cell lines exhibited a dose-dependent increase in cell death in EAAT3-silenced cells (Fig. 4D). It is reasonable to conclude that cells silenced for P65 exhibited even fewer live cells, displaying greater sensitivity to ferroptosis induced by RSL3 treatment, with a more pronounced trend (Fig. 4E). To further confirm RSL3-induced ferroptosis in EAAT3 and P65 knockdown cell lines, we performed TEM analysis, a classical imaging technique for detecting ferroptosis-related morphological changes. As shown in Fig. 4F, in si-EAAT3 and si-P65 cells, despite being in a state of oxidative stress, TEM revealed that mitochondrial morphology remained largely normal, with only a slight increase in electron density and occasional mitochondrial membrane rupture, which appeared to be within the cells’ adaptive capacity. However, following RSL3 treatment, TEM revealed pronounced mitochondrial shrinkage, significantly increased electron density, extensive membrane rupture, and loss of continuity, collectively indicating mitochondrial dysfunction and ferroptosis (Fig. 4F). Using the FerrDb database, we analyzed the GEO dataset GSE44619. The differentially expressed genes related to ferroptosis and suppression displayed varied expression patterns after NF-κB inhibition (Fig. 4G and Supplementary Fig. 5B). Moreover, ROS-related hypoxia genes also exhibited differing expression patterns in this dataset (Supplementary Fig. 5C). To further strengthen this relationship, we treated cells with the NF-κB inhibitor QNZ. As shown, the ferroptosis marker ACSL4 was induced in a dose-dependent manner with QNZ treatment, while SLC7A11 was inhibited in the A549 cell line (Fig. 4H). Intriguingly, GPX4, which protects cells against membrane lipid peroxidation, was induced after QNZ treatment (Fig. 4H). This observation aligns with the rationale that RSL3 is a drug that inhibits GPX4 and thus, enhances the sensitivity of cells to ferroptosis. An increase in GPX4 expression in tumor cells can make them more susceptible to ferroptosis-inducing agents. As mentioned earlier, NF-κB inhibition increases ROS levels. Therefore, we employed GSH-MEE to neutralize ROS generation. Following GSH-MEE treatment, the ROS marker CHAC1 decreased in NF-κB inhibition cells, and GPX4, the ferroptosis marker, was also rescued (Fig. 4I), although ACSL4 showed no significant change. These findings collectively indicate that inhibiting NF-κB/EAAT3 can increase sensitivity to ferroptosis, particularly when induced by the ferroptosis inducer RSL3.

### NF-κB induces EAAT3 expression via two putative cis-elements in its promoter

**Fig. 5:**
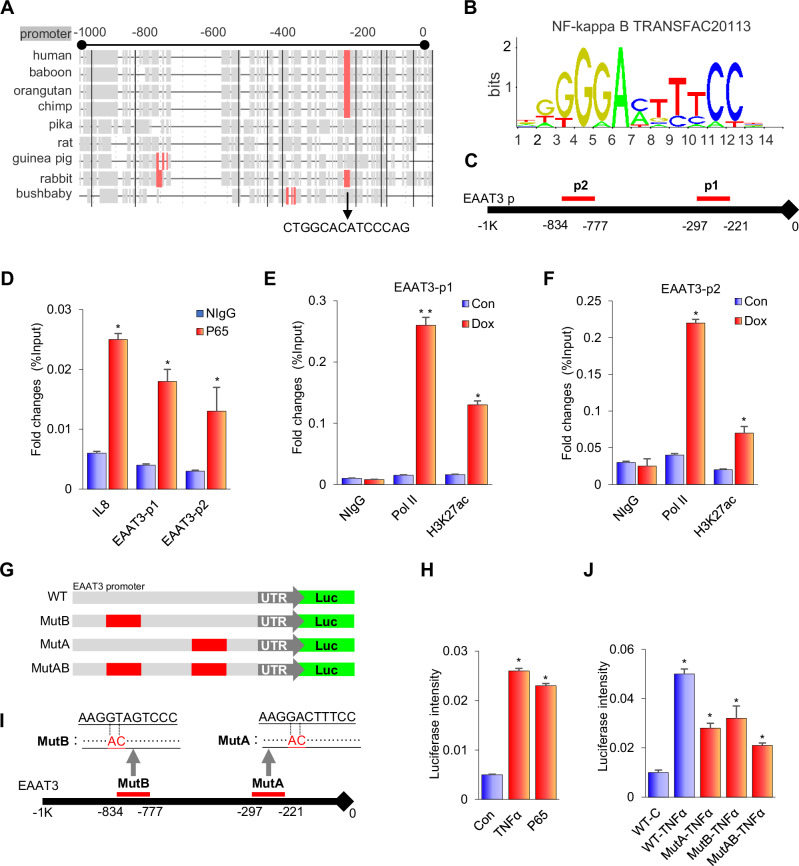
NF-κB induces EAAT3 expression via two putative cis-elements in its promoter. **A** The Contra V3 website analysis of the conservation region in EAAT3 promoter. **B**,**C** Two putative P65 binding motifs (p1 and p2) were predicted via ALGGEN and JARSPAR websites in the promoter of EAAT3. **D** ChIP analysis: IP of P65 followed by PCR of the sequences in the promoter of EAAT3. N IgG was negative control, and the IL-8 promoter was positive control. **E**,**F** ChIP analysis: IP of RNA Poly II and H3K27ac followed by PCR of the sequences in the promoter of EAAT3. N IgG was negative control, and the IL-8 promoter was positive control. **G**,**I** The design sketch of EAAT3 promoter-derived luciferase plasmid and the mutation region (MutA and MutB). **H** The promoter of EAAT3-derived luciferase was detected after TNFα treatment or transfecting the P65 plasmid. **J** The promoter of EAAT3-derived luciferase (WT, MutA, MutB, and MutAB) was detected after TNFα treatment. *p < 0.05, **p < 0.01, error bars represent SEM.

NF-κB is a transcription factor that plays a central role in mediating inflammatory signaling. The aforementioned observations prompt the question of whether NF-κB directly regulates EAAT3 expression at the transcriptional level. To investigate whether NF-κB directly activates EAAT3 transcription, we analyzed the DNA sequence of the EAAT3 promoter for potential NF-κB binding cis-elements using The Contra V3 software and the JASPAR website (Fig. 5A, B). The analysis revealed the presence of two candidate NF-κB binding sites in the promoter of EAAT3 (Fig. 5C). To determine if NF-κB could directly bind to these sites, we conducted ChIP analysis with P65 immunoprecipitation and PCR sequencing in the EAAT3 promoter. The P65 subunit of NF-κB was indeed found to be bound to the two putative NF-κB binding motifs within the EAAT3 promoter. As a negative control, normal IgG did not bind to the promoter, and as a positive control, P65 was shown to bind to the IL-8 promoter (Fig. 5D). Furthermore, RNA polymerase II and H3K27ac were also recruited to the EAAT3 promoter in cells with overexpressed exogenous P65 (Dox-induced myc-tagged-p65 expression) (Fig. 5E, F and Supplementary Fig. 6A). Additionally, we performed a bioinformatic analysis of GSE160856. In this P65 ChIP-Seq data, we observed that P65 was recruited to the EAAT3 promoter, particularly near the transcription start site (TSS) region (Supplementary Fig. 6B).

To determine if these sites could mediate transcription in response to NF-κB stimulation, we constructed an EAAT3 promoter-derived luciferase reporter plasmid, WT-luc (Fig. 5G). Transfection of HEK293T cells with this WT-luc construct, either alone or in combination with P65, or in treating cells with TNFα, resulted in the activation of luciferase activity (Fig. 5H). This indicates that the EAAT3 promoter is a direct target of NF-κB signaling. To elucidate the specific role of each putative binding site, we generated constructs with mutations in the P65 binding motif 1 (MutA), motif 2 (MutB), or both (MutAB) (Fig. 5G–I). We then performed luciferase reporter analyses by transfecting HEK293T cells with these constructs, either alone or in combination with P65, or by treating the cells with TNFα. Co-transfection of cells with the EAAT3 promoter-driven luciferase reporters, specifically WT-luc, followed by treatment with TNFα or co-transfection with P65, led to the activation of luciferase activity with the wild-type EAAT3 promoter (WT-TNFα or WT-P65). However, the TNFα or P65-mediated activation was significantly reduced when using the EAAT3 promoter-derived luciferase reporter with a single motif mutation, either Mut A or Mut B. Moreover, the activation was completely abolished when using the double motif-mutated luciferase plasmid, MutAB (Fig. 5J and Supplementary Fig. 6C). Hence, it is evident that both putative P65 binding motifs in the EAAT3 promoter are essential for NF-κB-induced activation.

### EAAT3 is upregulated in a subset of NSCLC tissues and positively correlated with P65

**Fig. 6:**
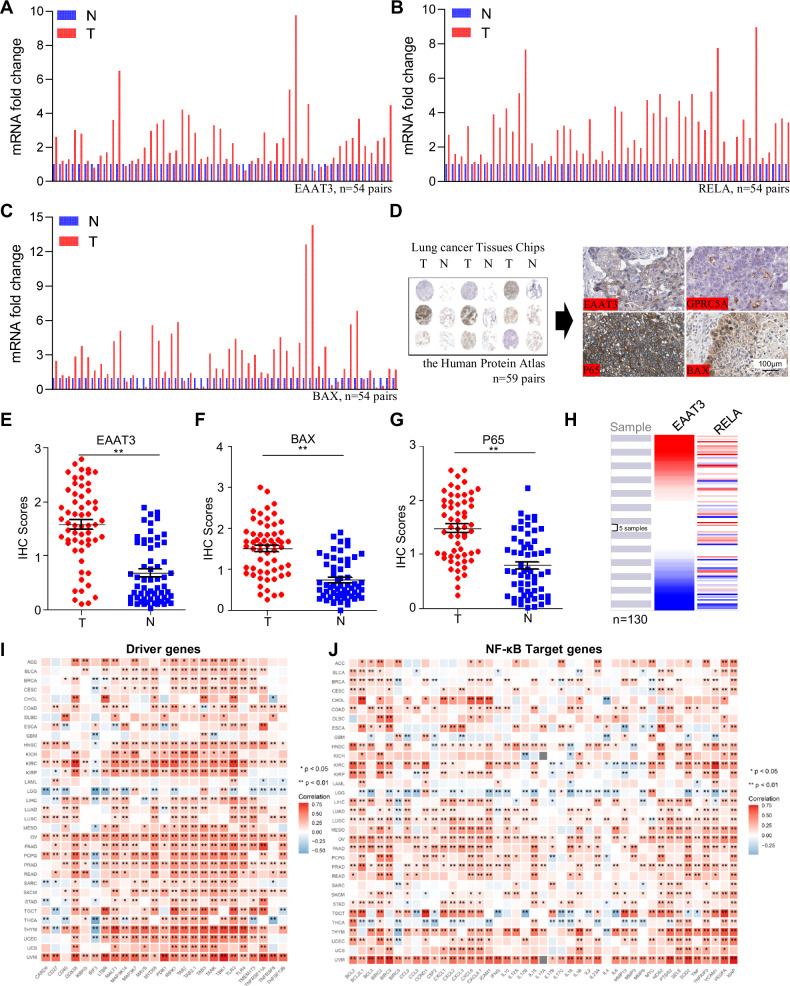
EAAT3 is upregulated in a subset of NSCLC tissues and positively correlated with P65. **A**–**C** Relative mRNA expression of EAAT3, RELA, and BAX in a set of human lung tumors compared with adjacent normal lung tissues (*n* = 54 pairs). **D** The expression of EAAT3, GPRC5A, P65, and BAX by IHC staining in a human lung tissue chip that includes tumors and adjacent normal tissues from The Human Protein Atlas datasets (*n* = 59 pairs). **E**–**G** The IHC scores of the expression EAAT3, BAX, and P65. **H** UCSC Xena database showed the relationship between EAAT3 and RELA. **I**,**J** Different gene expressions of TCGA pan-cancer show the relationship between EAAT3 and NF-κB driver and target genes. *p < 0.05, **p < 0.01, error bars represent SEM.

To investigate the potential correlation between EAAT3 expression and NF-κB signaling, we conducted qPCR analysis on 54 pairs of NSCLC tissues and their respective adjacent normal tissues. Our findings reveal a significant association, with elevated EAAT3 levels prevalent in 47 out of 54 NSCLC samples, coinciding with increased expressions of RELA and the NF-κB signaling target gene, BAX (Fig. 6A–C). To comprehensively elucidate this expression correlation, we performed immunohistochemistry (IHC) analyses using data sourced from The Human Protein Atlas, consisting of 59 paired samples available at https://www.proteinatlas.org/ (Fig. 6D). Remarkably, EAAT3, P65, and BAX exhibited pronounced expression in lung tumor tissues compared to their adjacent normal counterparts. Conversely, GPRC5A displayed heightened expression predominantly within the adjacent normal tissues (Fig. 6D). The IHC staining scores further demonstrated a robust correlation within clinical NSCLC samples (Fig. 6E–G and Supplemental Fig. 7A). Moreover, we corroborated our findings using the publicly accessible UCSC Xena database (https://xenabrowser.net/) to validate the relationship between EAAT3 and RELA. Within the dataset (Raponi 2006) *n* = 130 instances of lung cancer with elevated RELA expression corresponded to similarly heightened EAAT3 expression (Fig. 6H). Leveraging TCGA RNA sequencing data, subjected to analysis through TIMER2.0 (http://timer.cistrome.org/), we observed a positive correlation between EAAT3 and RELA, as well as NF-κB target genes such as BAX and BCL2 (Supplemental Fig. 7B–D). Interestingly, the TCGA pan-cancer relationship analysis showed that the expression of EAAT3 positively correlated with the expression of NF-κB driver genes and target genes in most cancer types (Fig. 6I, J). Collectively, these extensive datasets substantiate our hypothesis that increased NF-κB activity potentially contributes to the upregulation of EAAT3, which may not be limited to lung cancer.

### The inflammation-inducing factor, smoking, increased the expression of EAAT3

**Fig. 7:**
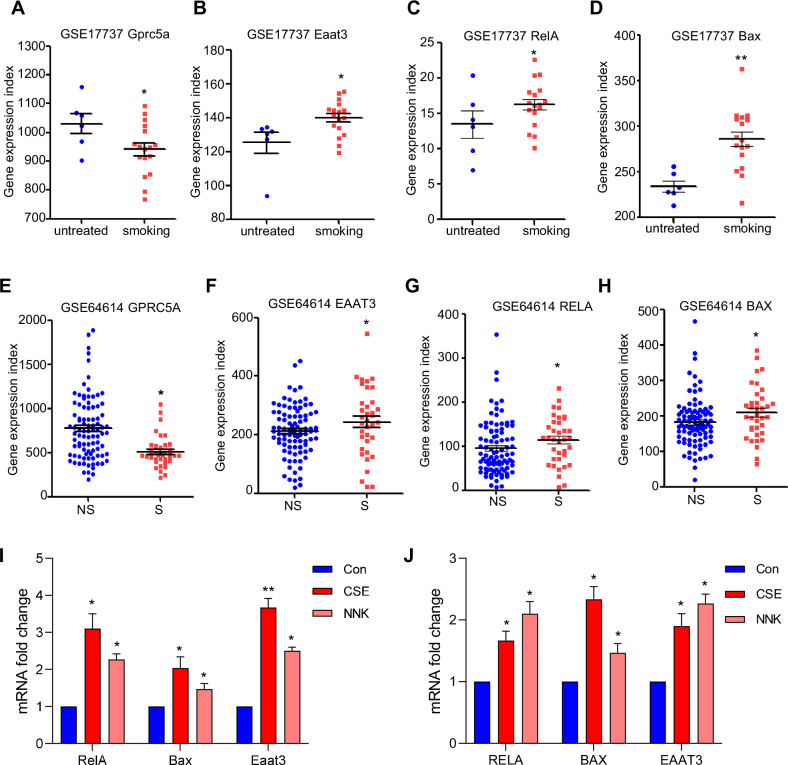
The inflammation-inducing factor, smoking, increased the expression of EAAT3. **A**–**D** GSE17737 data from the GEO database showed the expression of Gprc5a, Eaat3, RelA, and Bax in the untreated group and the smoking group, analyzed with GraphPad Prism 5. **E**–**H** GSE64614 data from GEO database showed the expression of GPRC5A, EAAT3, RELA, and EAAT3 in smokers compared with non-smoker patients, analyzed with GraphPad Prism 5. **I** The mRNA expression of RelA, Bax and Eaat3 was induced following treatment with CSE and NNK (0.5 µg/ml) in MTEC cells. **J** The mRNA expression of RELA, BAX and EAAT3 was induced following treatment with CSE and NNK (0.5 µg/ml) in 16HBE cells. *p < 0.05, **p < 0.01, error bars represent SEM.

Given that the NF-κB inflammatory pathway is involved in the regulation of EAAT3 expression, we sought to gather supporting data from the GEO database. As illustrated in GSE17737, mice received nose-only exposure to 4% mainstream cigarette smoke or air for 2 h/day, 5 days/week over 12 weeks. Mice exposed to smoke, a known inducer of inflammation, exhibited suppressed Gprc5a expression and increased Eaat3 and RelA (Fig. 7A–C). The target gene of NF-κB signaling, Bax, also displayed elevated expression in the smoking group, further correlating with EAAT3 (Fig. 7D). In another dataset (GSE64614), the expression of identified distal and proximal signatures in the small airway epithelium (SAE) of smokers with COPD was compared to that of healthy nonsmokers. The statistics showed that the expression of EAAT3 was enhanced in smokers with COPD compared to healthy nonsmokers, while GPRC5A was correspondingly suppressed in smokers (Fig. 7E, F) [40]. Furthermore, NF-κB signaling-related genes RELA and BAX exhibited increased expression in smokers (Fig. 7G, H). To further validate this observation, we treated MTEC and 16HBE separately with cigarette smoke extract (CSE) and garlic oil, as well as 4-(methylnitrosamino)-1-(3-pyridyl)-1-butanone (NNK), a compound also known as a key carcinogen in tobacco. Both CSE and NNK, major inflammatory inducers in the smoking process, activated the NF-κB pathway, as evidenced by the increased mRNA expression of RELA and its target gene BAX (Fig. 7I, J). Interestingly, EAAT3 mRNA levels were also upregulated following CSE and NNK stimulation (Fig. 7I, J). A potential mechanism for this induction might be that the induced EAAT3 enhances GSH production, helping cells combat oxidative stress. Collectively, these data support the notion that increased GSH synthesis via NF-κB-mediated EAAT3 upregulation serves to counteract the high levels of ROS in smokers.

## Discussion

In this study, we have demonstrated the critical role of increased GSH synthesis in countering oxidative stress during lung tumorigenesis, utilizing the Gprc5a^−/−^ mouse model. Our findings highlight how dysregulated glutamate transporter EAAT3 enhances glutamate uptake, thereby facilitating cystine uptake and GSH synthesis. This enhancement of GSH production also augments the stemness-like activities of cancer cells.

Furthermore, we have identified two putative P65 binding sites in the EAAT3 promoter that are responsible for NF-κB-mediated induction of EAAT3. Inhibition of NF-κB/EAAT3 leads to increased ROS generation and heightened sensitivity to ferroptosis induced by RSL3. Lastly, we have shown that glutamate transporters, including EAAT3, are upregulated in the majority of NSCLC samples. This upregulation of glutamate transporters enhances glutamate uptake, thus facilitating cystine uptake and GSH synthesis, ultimately contributing to lung tumor development.

Inflammation has been correlated with a heightened production of ROS. For instance, TNFα, primarily generated by activated macrophages, has the potential to increase ROS production in lung epithelial cells, which is a plausible mechanism for inflammation-induced carcinogenesis [41]. As we previously demonstrated, lung tumorigenesis in Gprc5a^−/−^ mice is associated with chronic inflammation. Therefore, it is expected that ROS production is elevated under conditions of inflammation and tumor development. To counterbalance the elevated ROS levels, malignant cells must acquire the ability to generate a significant amount of GSH. This increase in GSH levels results from metabolic reprogramming since the standard GSH synthesis system, under normal physiological conditions, may prove inadequate for the demands of tumorigenesis [42].

In neurons, the primary role of the glutamate transporter EAAT3 is to facilitate the uptake of extracellular glutamate in the central nervous system. This function is critical for maintaining precise neurotransmission, as it helps regulate extracellular glutamate concentrations to prevent excitotoxic damage. EAAT3 is also integral to glutathione synthesis in neurons. Studies with Eaat3-knockout mice have demonstrated that the absence of Eaat3 leads to reduced glutathione content in neurons and early onset brain aging due to oxidative stress. Treatment with the antioxidant N-acetyl-cysteine significantly improves neuronal function in these mice [43]. Glutamate uptake through EAAT3 is closely linked to the activation of system X_c_^–^. System X_c_^–^ imports cystine while exporting glutamate in a 1:1 ratio. Extracellular glutamate acts as a competitive inhibitor for cystine uptake via system X_c_^–^. Therefore, EAAT3 not only transports extracellular glutamate but also contributes to the intracellular glutamate pool required for cystine import. In fact, transient overexpression of EAAT3 in hippocampal HT22 cells led to increased intracellular GSH levels in the presence of high glutamate concentrations, offering protection against oxidative glutamate toxicity [44, 45]. These effects were particularly pronounced when EAAT3 was co-overexpressed with xCT [46]. In our current study, we have observed that both xCT and EAAT3 are overexpressed in lung tumors that develop in *Gprc5a*-ko mice and in a subset of NSCLC tissues. These findings suggest that lung tumor cells have co-opted the system X_c_^–^ and EAAT3, a system typically associated with neurons, to facilitate GSH production. While cystine is generally considered the rate-limiting amino acid for GSH synthesis [47], glutamate also plays a crucial role in GSH production, especially in cancer cells with a high demand for GSH. For cancer cells lacking upregulated EAAT3, it is plausible that other glutamate transporter systems are elevated, and further investigation is required to confirm this. Nevertheless, it is clear that the uptake of glutamate and the synthesis of GSH must be maintained at high levels for cancer development.

NF-κB is a redox-regulated sensor that responds to oxidative stress and can be activated by low doses of hydrogen peroxide [48, 49]. In our study, we established a connection between NF-κB and the regulation of EAAT3. Our analysis has identified two potential NF-κB binding sites in the EAAT3 promoter, indicating that this gene is regulated by signaling pathways associated with inflammation. Significantly, we have observed that the expression of EAAT3 is suppressed in Gprc5a-/-/SPC-dnIκB-α mice [37], underscoring the requirement for NF-κB in the upregulation of EAAT3. This regulatory pattern is similar to what has been observed with xCT, as previous studies have reported that xCT expression can be induced by inflammatory stimuli, including substances like LPS and TNFα. Furthermore, there is a putative NF-κB binding site in the promoter of xCT [50]. While our findings indicate that NF-κB can specifically bind to EAAT3 promoter regions in certain cell types, multiple pathways and factors are known to regulate EAAT3, including the NRF2-ARE pathway [51], the RFX1 transcription factor [52], NFAT5/TonEBP [53], and ATRA [54]. Therefore, NF-κB is one of several transcription factors capable of binding to the promoter and activating EAAT3 expression. Besides, several exogenous factors have been shown to activate EAAT3, primarily by influencing NF-κB signaling, which subsequently upregulates EAAT3 expression. Key factors include: (1) Pro-inflammatory cytokines: Cytokines such as TNFα and interleukin-1 beta (IL-1β) are known to activate NF-κB, leading to increased EAAT3 expression. (2) Oxidative stress: Due to rapid proliferation and metabolic shifts, lung cancer cells experience high levels of oxidative stress, which activates NF-κB. This activation upregulates EAAT3 as part of the cell’s defense against ROS. (3)Hypoxia: Under hypoxic conditions, NF-κB activation enhances EAAT3 expression, strengthening antioxidant defenses in lung cancer cells. (4)Environmental toxins and pollutants: Exposure to pollutants and carcinogens, such as cigarette smoke and airborne particulate matter, can trigger NF-κB signaling in lung tissue, contributing to EAAT3 activation.

While our study has shed light on the link between inhibiting NF-κB/EAAT3, ROS accumulation, and increased ferroptosis sensitivity in lung cancer cells, the precise molecular mechanisms underlying this process remain unclear. Silencing P65 or EAAT3 increases ROS levels and reduces GSH synthesis, but these changes alone are insufficient to trigger significant cell death due to the robust ferroptosis-resistance mechanisms in cancer cells, including the xCT-GSH-GPX4 system and other protective pathways. This resistance allows cancer cells to tolerate ROS accumulation and maintain survival unless the balance is further disrupted by external ROS inducers (e.g., H_2_O_2_) or inhibitors of ferroptosis-resistance mechanisms (e.g., RSL3 and Erastin). We also have observed an increase in the expression of ACSL4 when cells are treated with an NF-κB inhibitor, which could explain why ferroptosis becomes more sensitive to RSL3. Additionally, the expression of SLC7A11 decreased, which is another factor contributing to ferroptosis sensitivity. However, it is worth noting that GPX4, an antioxidant enzyme glutathione peroxidase that protects cells against oxidative stress [55], was also increased when NF-κB was inhibited. This suggests that the cells rely on GPX4 to resist oxidative stress, and this could potentially increase their sensitivity to RSL3, a ferroptosis inducer that targets GPX4. While these findings provide valuable insights, we acknowledge that further research is needed to delve into the specific molecular mechanisms at play in this context.

## Supplementary information

Supplemental Figure 1

Supplemental Figure 2

Supplemental Figure 3

Supplemental Figure 4

Supplemental Figure 5

Supplemental Figure 6

Supplemental Figure 7

Supplemental Figure Legends

Western blot no-cut Raw data

## Data Availability

The data will be provided by the authors upon reasonable request.
