# Correction: NF-κB-mediated EAAT3 upregulation in antioxidant defense and ferroptosis sensitivity in lung cancer

**DOI:** 10.1038/s41419-025-07518-y

**Published:** 2025-04-03

**Authors:** Donghua Wen, Wenjing Li, Xiang Song, Min Hu, Yueling Liao, Dongliang Xu, Jiong Deng, Wenzheng Guo

**Affiliations:** 1 Department of Laboratory Medicine, Shanghai East Hospital, Tongji University School of Medicine, Shanghai, 200120 China; 2 Breast Cancer Center, Shandong Cancer Hospital and Institute, Shandong First Medical University and Shandong Academy of Medical Sciences, Jinan, Shandong 250117 China; 3 College of Life and Environmental Science, Wenzhou University, Wenzhou, 325035 China; 4 Renji Hospital, Shanghai Jiao Tong University School of Medicine, Shanghai, 200025 China; 5 Medical Research Center, Affiliated Hospital of Binzhou Medical University, Binzhou, 256600 Shandong China

**Keywords:** Non-small-cell lung cancer, Stress signalling

Correction to: *Cell Death and Disease* 10.1038/s41419-025-07453-y, published online 22 February 2025

In this article the designation of co-first authorship is missing.

The correct author contribution statement should be: “Donghua Wen, Wenjing Li, Xiang Song, and Min Hu contributed equally to this work.”

The original article has been corrected.

