# Supplementary figures and images for: NF-κB-mediated EAAT3 upregulation in antioxidant defense and ferroptosis sensitivity in lung cancer
Source: Cell Death Dis. 2025 Feb 22;16(1):124. doi: 10.1038/s41419-025-07453-y (PMC11847022; doi:10.1038/s41419-025-07453-y)

## Protein digestion and absorption

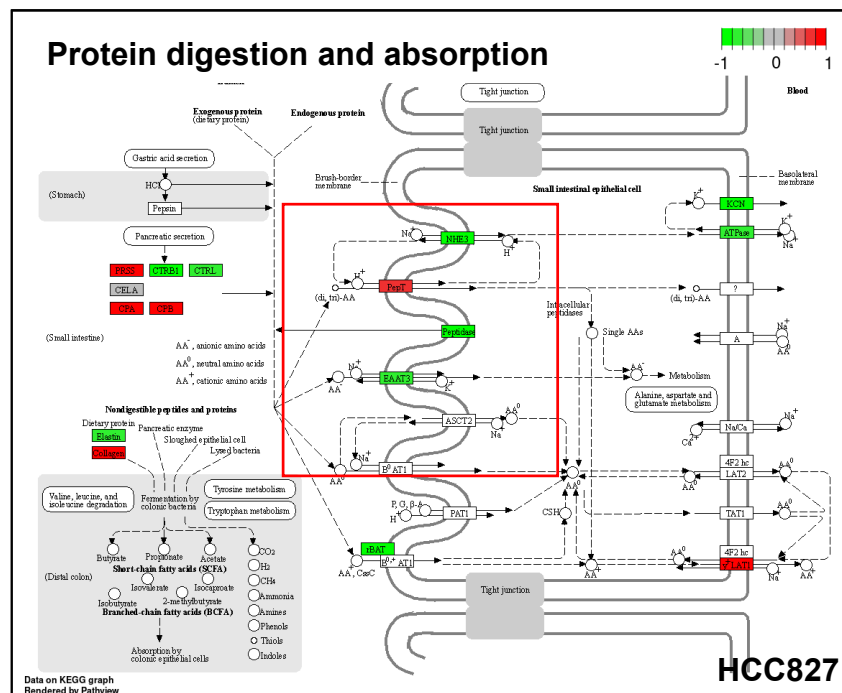

**B**

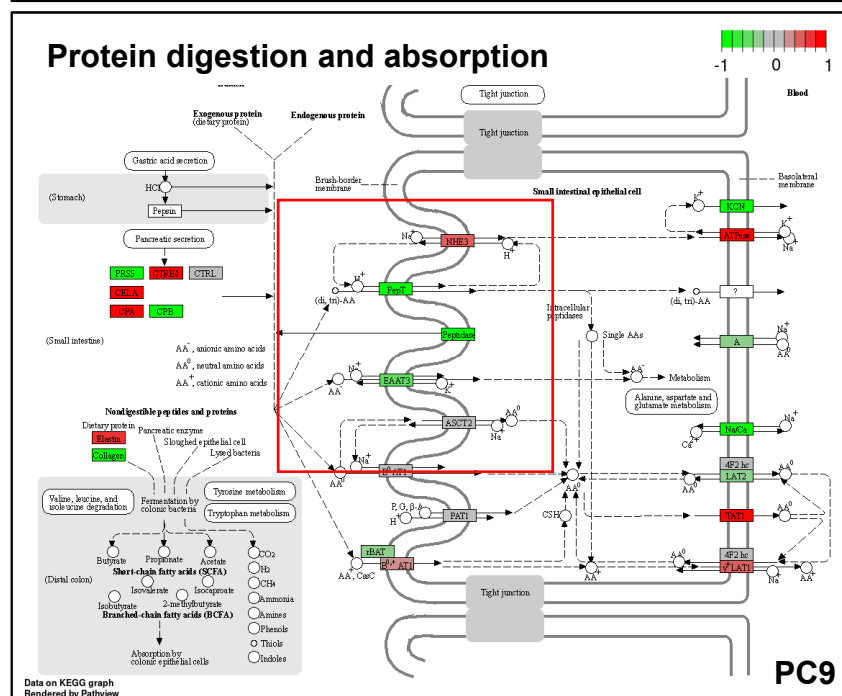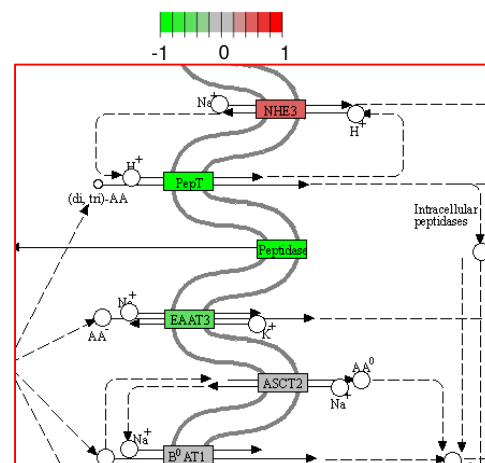

**C**

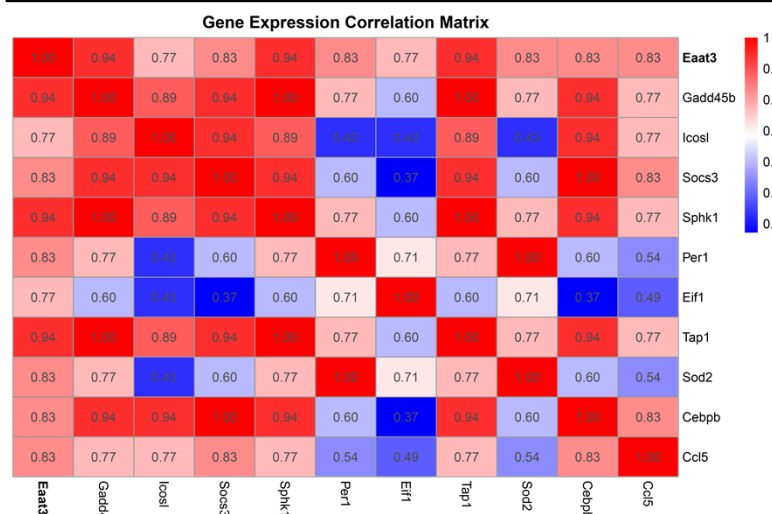

Relate to Fig 1

Supplement: Supplementary file 1 — Supplemental Figure 1 [file 41419_2025_7453_MOESM1_ESM.pdf]

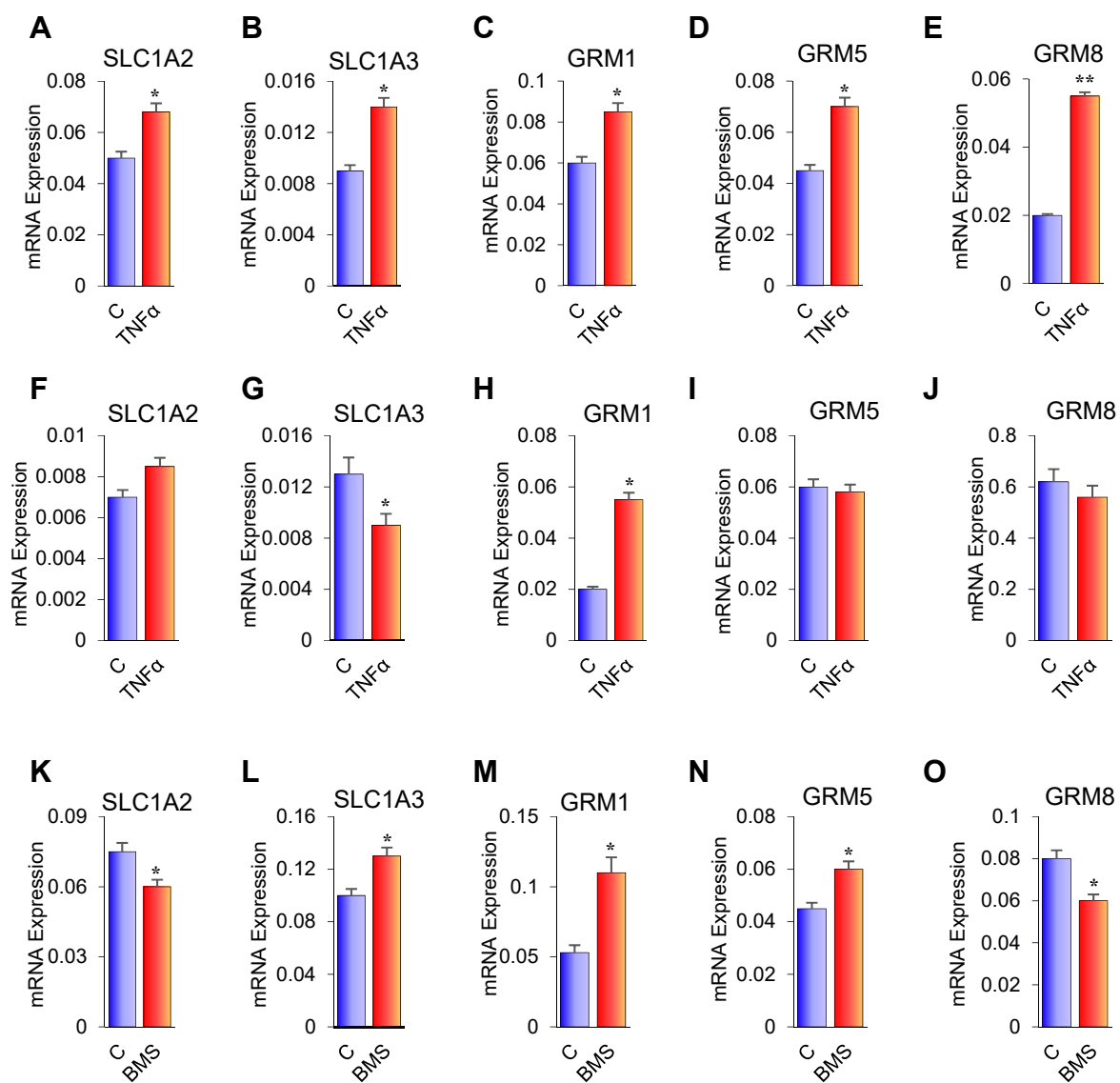

Relate to Fig 2

Supplement: Supplementary file 2 — Supplemental Figure 2 [file 41419_2025_7453_MOESM2_ESM.pdf]

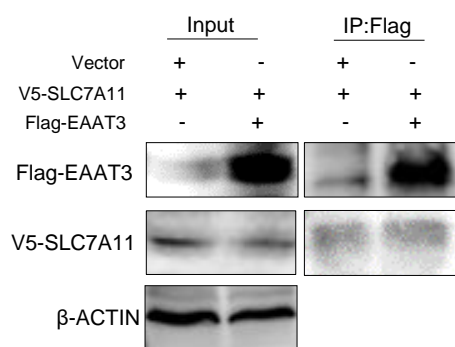

Relate to Fig 3

Supplement: Supplementary file 3 — Supplemental Figure 3 [file 41419_2025_7453_MOESM3_ESM.pdf]

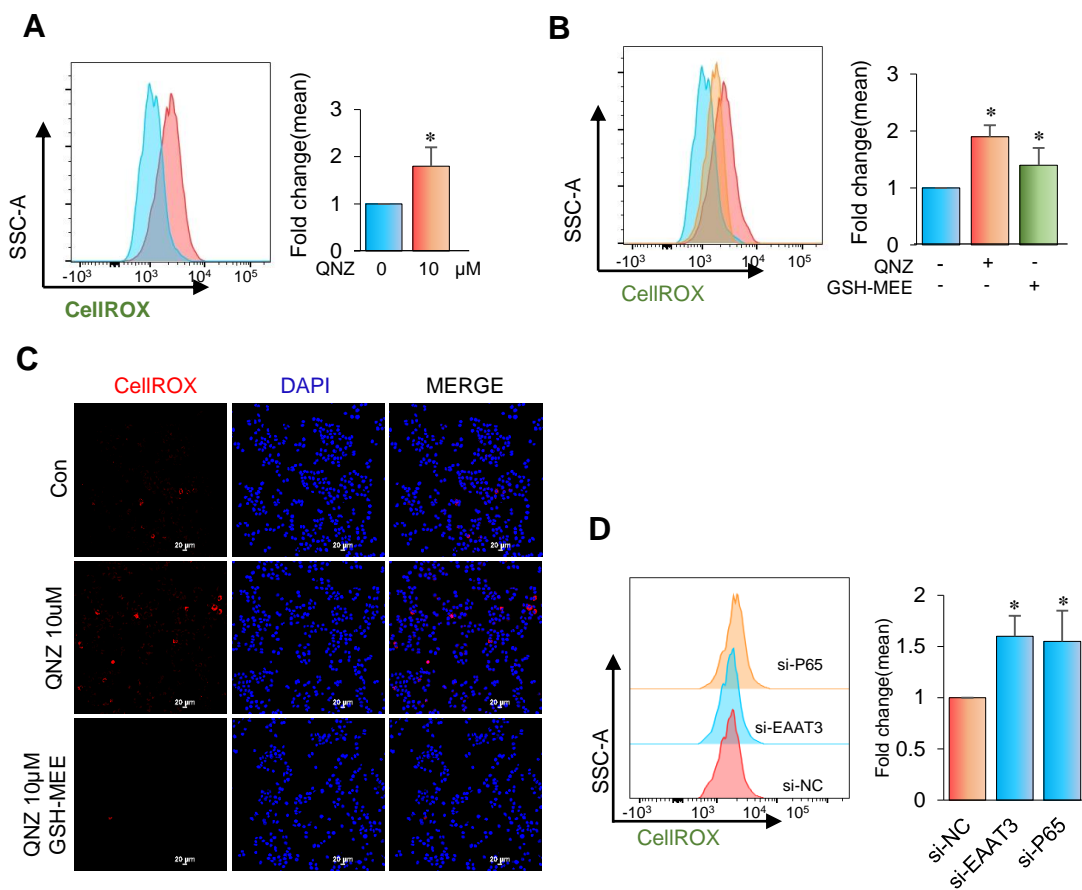

Relate to Fig 3

Supplement: Supplementary file 4 — Supplemental Figure 4 [file 41419_2025_7453_MOESM4_ESM.pdf]

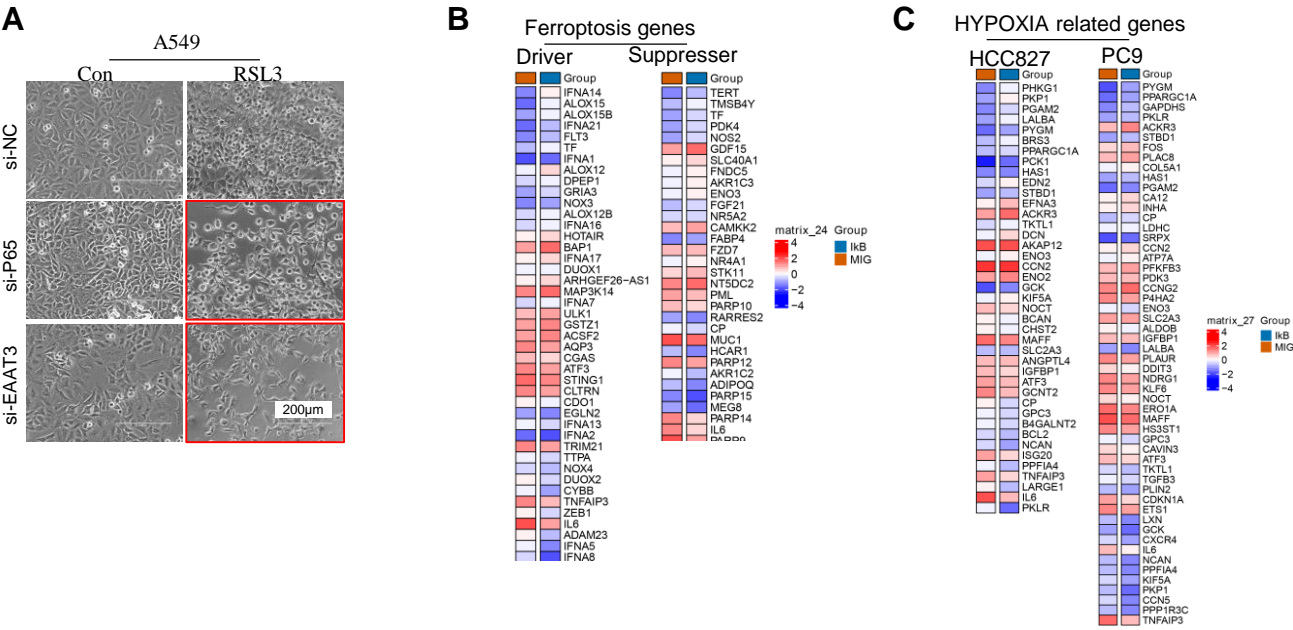

Relate to Fig 4

Supplement: Supplementary file 5 — Supplemental Figure 5 [file 41419_2025_7453_MOESM5_ESM.pdf]

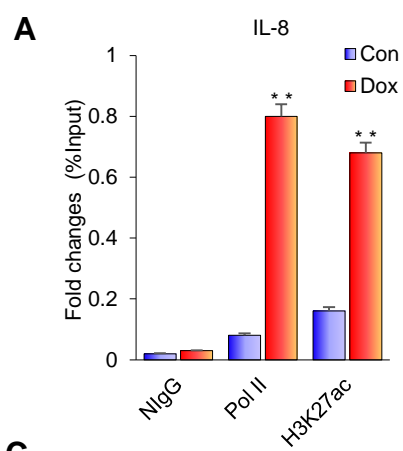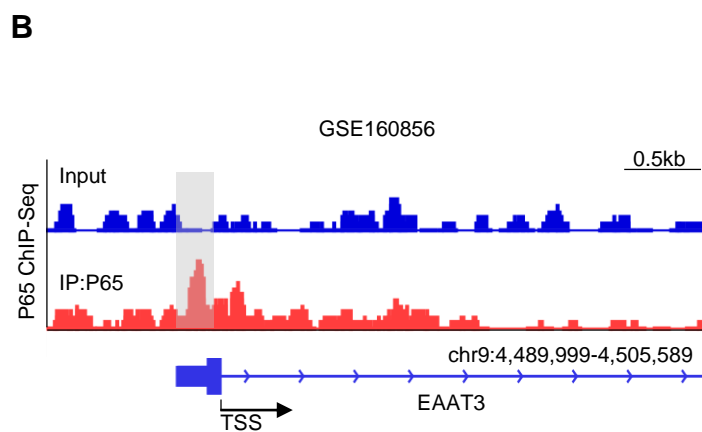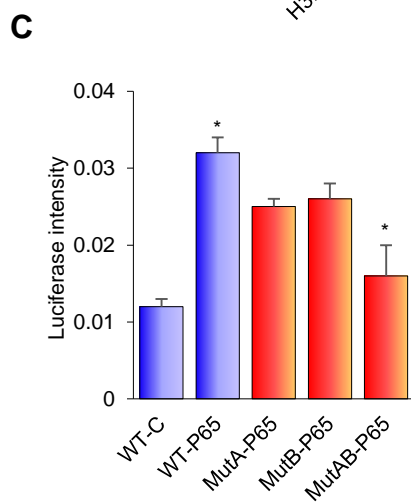

Relate to Fig 5

Supplement: Supplementary file 6 — Supplemental Figure 6 [file 41419_2025_7453_MOESM6_ESM.pdf]

**A**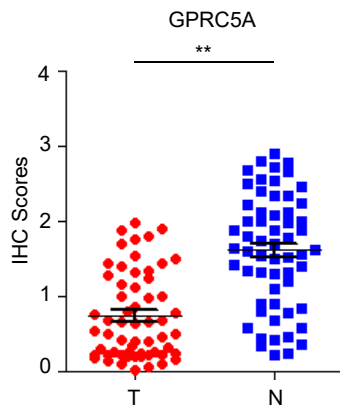**B**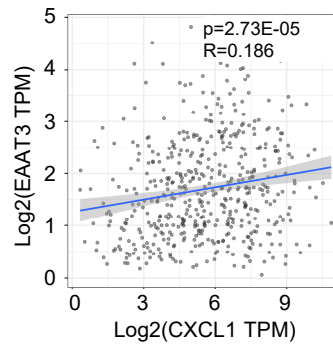**C**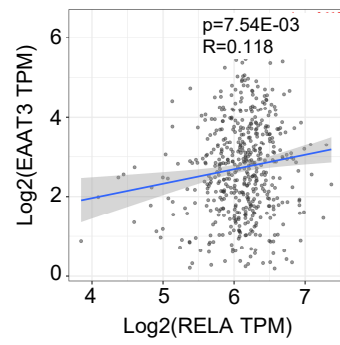**D**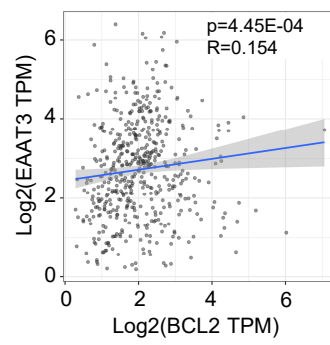

Relate to Fig 6

Supplement: Supplementary file 7 — Supplemental Figure 7 [file 41419_2025_7453_MOESM7_ESM.pdf]
