# Supplementary material for: NF-κB-mediated EAAT3 upregulation in antioxidant defense and ferroptosis sensitivity in lung cancer
Source: Cell Death Dis. 2025 Feb 22;16(1):124. doi: 10.1038/s41419-025-07453-y (PMC11847022; doi:10.1038/s41419-025-07453-y)
Supplement: Supplementary file 8 — Supplemental Figure Legends [file 41419_2025_7453_MOESM8_ESM.docx]

**NF-κB-Mediated EAAT3 Upregulation in Antioxidant Defense and Ferroptosis Sensitivity in Lung Cancer**

Donghua Wen^1,#^, Wenjing Li^1,#^, Xiang Song^2,#^, Min Hu^1, #^, Yueling Liao^3^, Dongliang Xu^4^, Jiong Deng^5,*^, Wenzheng Guo^1,*^

^1^ Department of Laboratory Medicine, Shanghai East Hospital, Tongji University School of Medicine, Shanghai, 200120, China.

^2^ Breast Cancer Center, Shandong Cancer Hospital and Institute, Shandong First Medical University and Shandong Academy of Medical Sciences, Jinan, Shandong 250117, China

^3^ College of Life and Environmental Science, Wenzhou University, Wenzhou, 325035, China

^4^ Renji Hospital, Shanghai Jiao Tong University School of Medicine, Shanghai, 200025, China

^5^ Medical Research Center, Affiliated Hospital of Binzhou Medical University, Binzhou 256600, Shandong, China.

^*^Correspondence to:

Wenzheng Guo, PhD (email: [wenzheng.guo@uky.edu](mailto:wenzheng.guo@uky.edu)), Shanghai East Hospital, Tongji University School of Medicine, Shanghai, 200120, China.;

Jiong Deng, PhD (email: [jiongdeng@bzmc.edu.cn](mailto:jiongdeng@bzmc.edu.cn)), Affiliated Hospital of Binzhou Medical University, Binzhou 256600, Shandong, China.

**Supplementary Figure Legends**

*Figure S1 NF-κB involved in the expression of EAAT3* **(A-B)** EAAT3 was enriched in the protein digestion and absorption pathway in HCC827 cell and PC9 cell. **(C)** RNA-seq shows the expression correlation between NF-κB pathway target genes and Eaat3 in KP model.

*Figure S2 SLC1A family members mRNA expression* **(A-E)** The mRNA expression of SLC1A2,SLC1A3,GRM1,GRM5 and GRM8 in human normal epithelial cell 16HBE with or without TNFα treatment. **(F-J)** The mRNA expression of SLC1A2,SLC1A3,GRM1,GRM5 and GRM8 in human normal epithelial cell HBEC with or without TNFα treatment. **(K-O)** The mRNA expression of SLC1A2,SLC1A3,GRM1,GRM5 and GRM8 in human ADC cell A549 with or without BMS treatment. * p<0.05, Error bars represent SEM.

*Figure S3 EAAT3 has no interaction with Xct* co-IP show that there is no interaction between EAAT3 and xCT(SLC7A11) in HEK293T cell.

*Figure S4 Inhibition of NF-κB increased the ROS* **(A)** FACS show the ROS level in PC9 cells treat with QNZ(10uM). **(B)** FACS show the ROS level in PC9 cells treat with QNZ (10uM) and GSH-MEE (2mM). **(C)** IF show the ROS level in PC9 cells treat with QNZ (10uM) and GSH-MEE (2mM). **(D)** FACS show the ROS level in PC9 cells silencing EAAT3 and P65 with siRNA. * p<0.05, Error bars represent SEM.

*Figure S5 Inhibition of NF-κB/EAAT3 increased ferroptosis sensitivity* **(A)** RSL3 (10uM) treatment induce more cell death in silenced P65 or silenced EAAT3 in A549 cell. **(B)** Different ferroptosis driver genes and suppresser genes expression in HCC827 cell from GSE44619. **(C)** Different Hypoxia related genes expression in PC9 and HCC827 cells from GSE44619.

*Figure S6 NF-κB induces EAAT3 expression via two putative cis-elements in its promoter* **(A)** ChIP analysis: IP of RNA Poly II and H3K27ac followed by PCR of the sequences in the promoter of EAAT3. N IgG was as negative control and the IL-8 promoter was positive control. **(B)** ChIP-Seq of GSE160856 visualization in area of EAAT3 promoter. **(C)** The luciferase was detected after mutating the p65 binding motif with transfecting p65 plasmid. * p<0.05, ** p<0.01, Error bars represent SEM.

*Figure S7 EAAT3 is up-regulated in a subset of NSCLC tissues and inversely correlated with p65* **(A)** IHC score show the GPRC5A expression level in tissue chip. **(B-D)** TIMER 2.0 dataset show the correlation between EAAT3 and CXCL1, RELA and BCL2 in lung cancer. ** p<0.01, Error bars represent SEM.
