# Supplementary material for: NF-κB-mediated EAAT3 upregulation in antioxidant defense and ferroptosis sensitivity in lung cancer
Source: Cell Death Dis. 2025 Feb 22;16(1):124. doi: 10.1038/s41419-025-07453-y (PMC11847022; doi:10.1038/s41419-025-07453-y)

Western blot no-cut Raw data

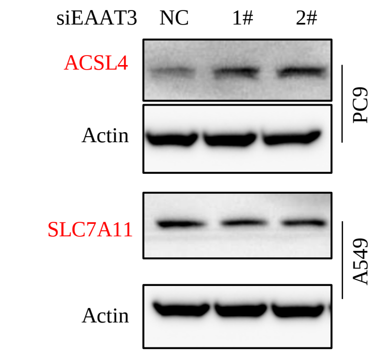
siEAAT3 NC 1# 2#
ACSL4
Actin
PC9
SLC7A11
Actin
A549

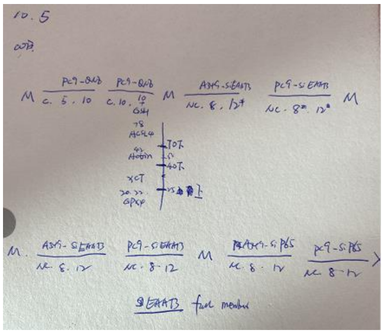

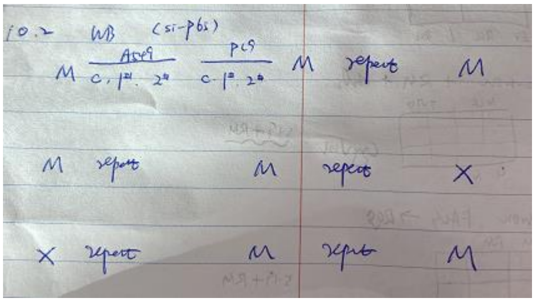

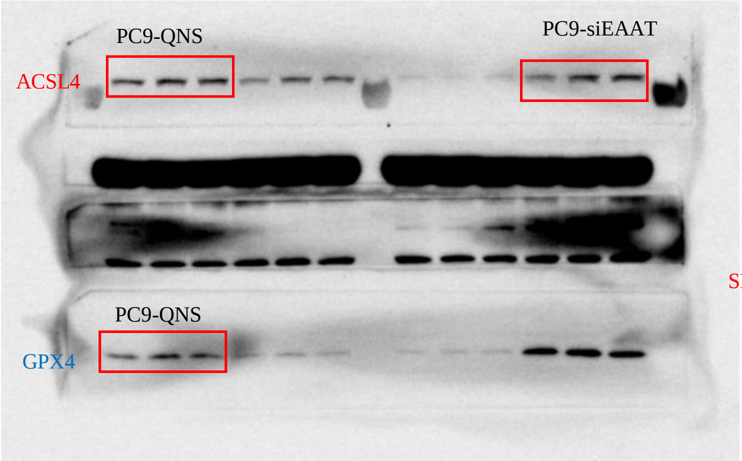
PC9-QNS
PC9-siEAAT
ACSL4
PC9-QNS
GPX4

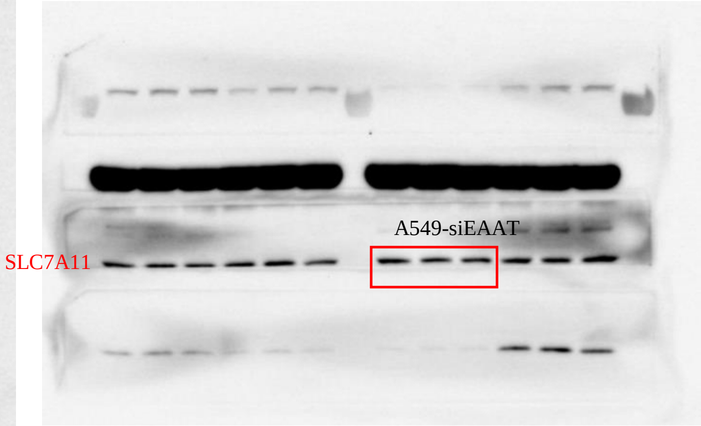
A549-siEAAT
SLC7A11

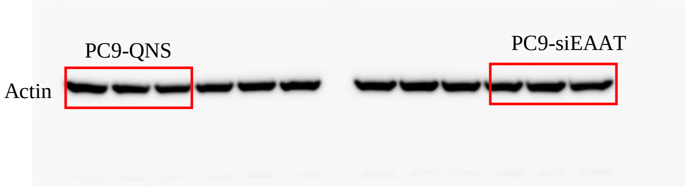
PC9-QNS
PC9-siEAAT
Actin

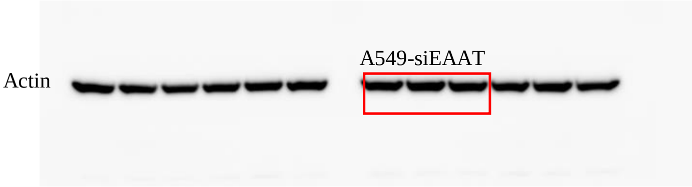
Actin
A549-siEAAT

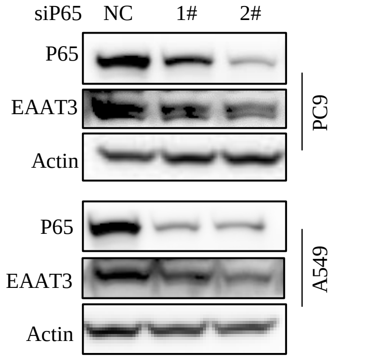
siP65 NC 1# 2#
P65
EAAT3
Actin
PC9
P65
EAAT3
Actin
A549

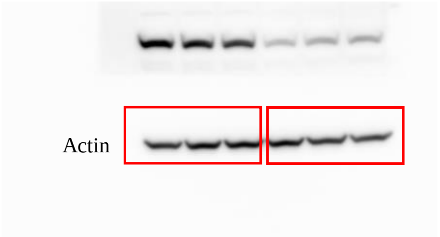
Actin

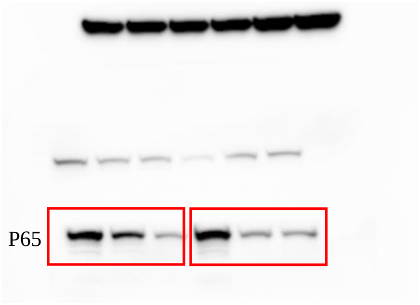
P65

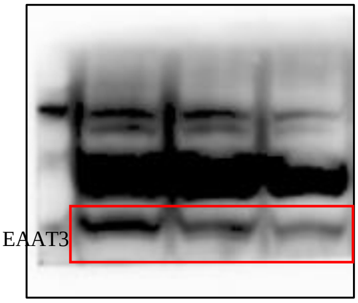
EAAT3

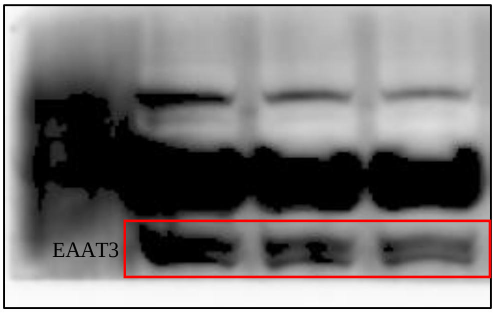
EAAT3

Original uncropped image

Fig 2

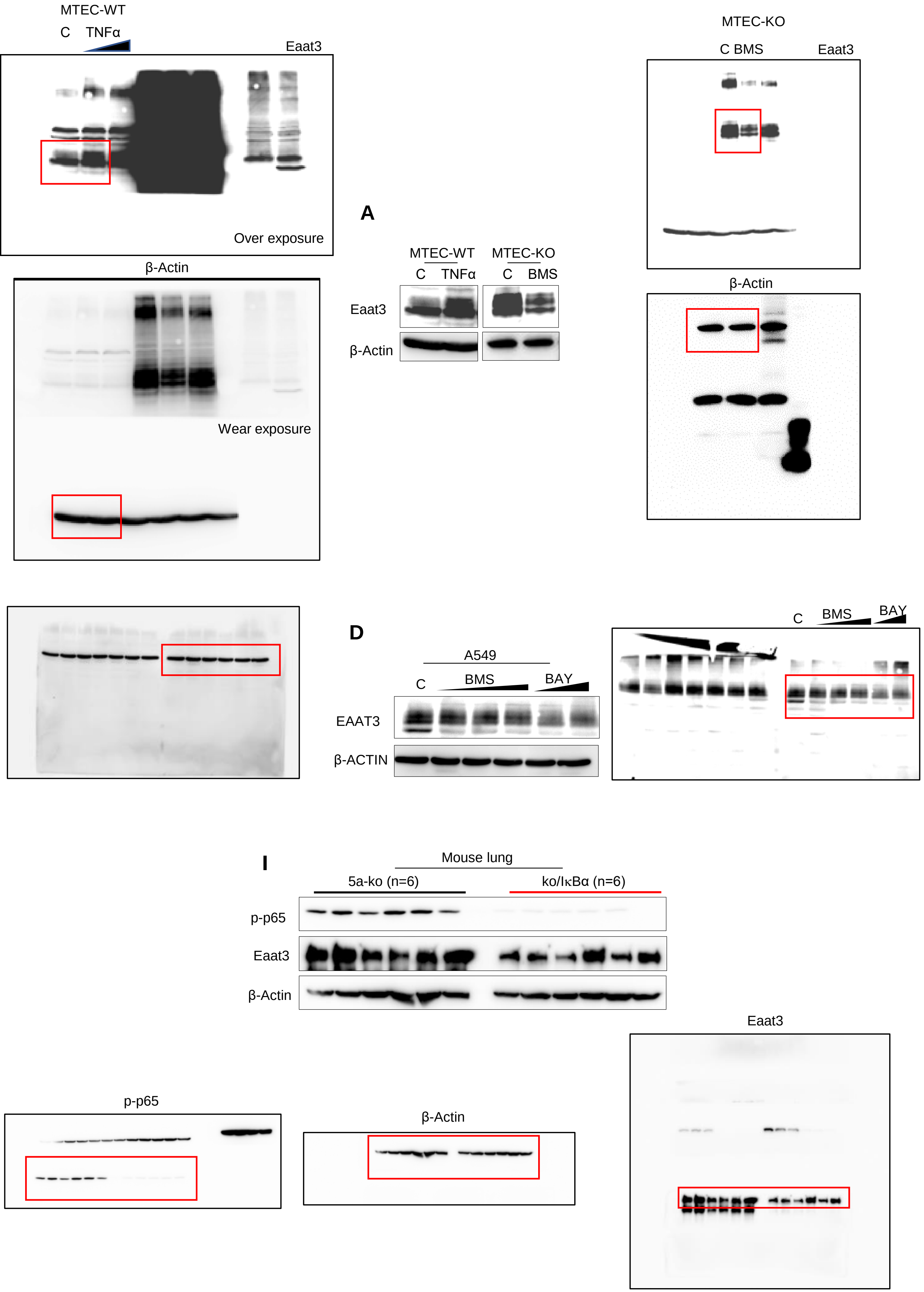

Original uncropped image

Fig 3

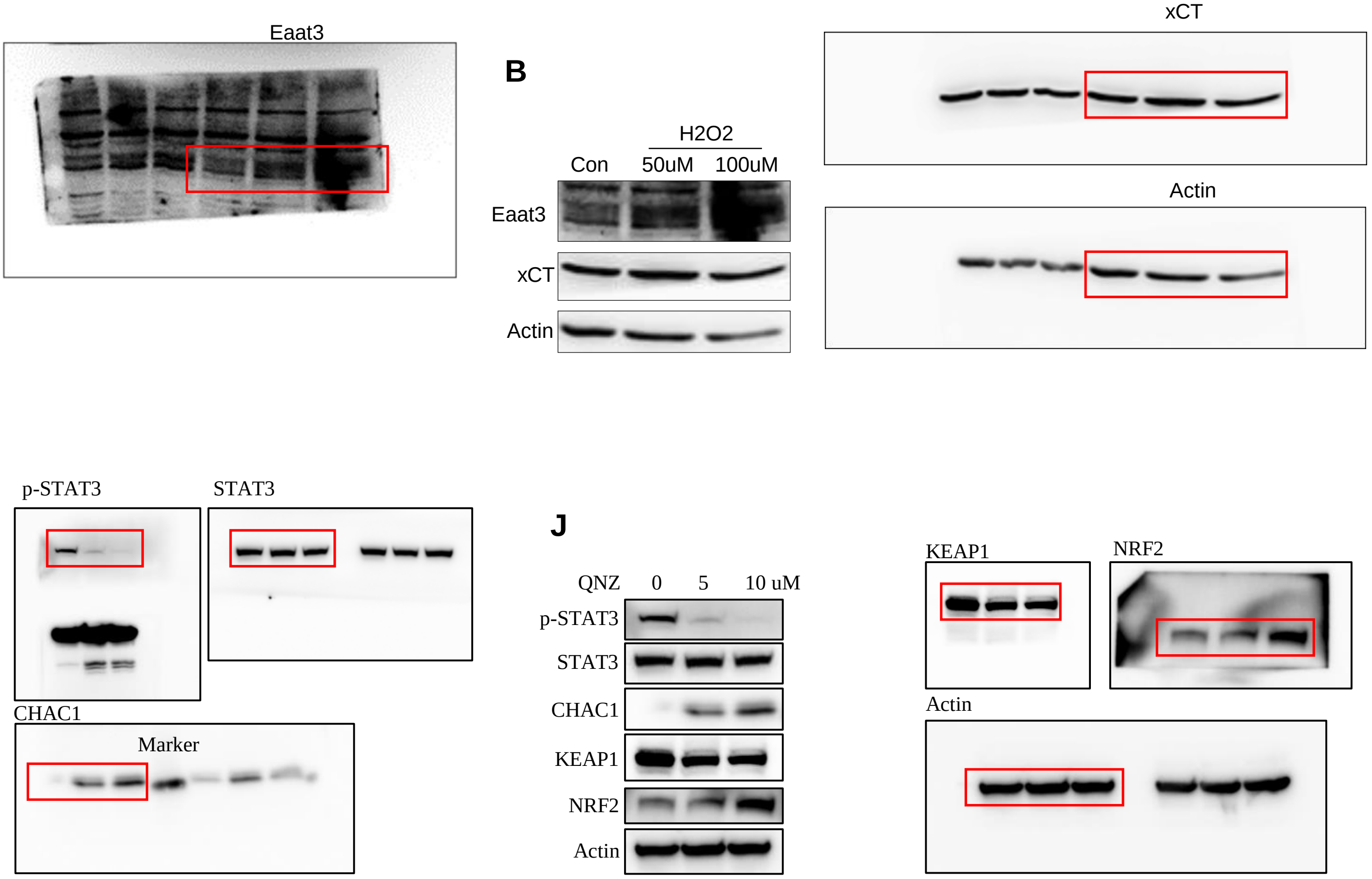

Fig 4

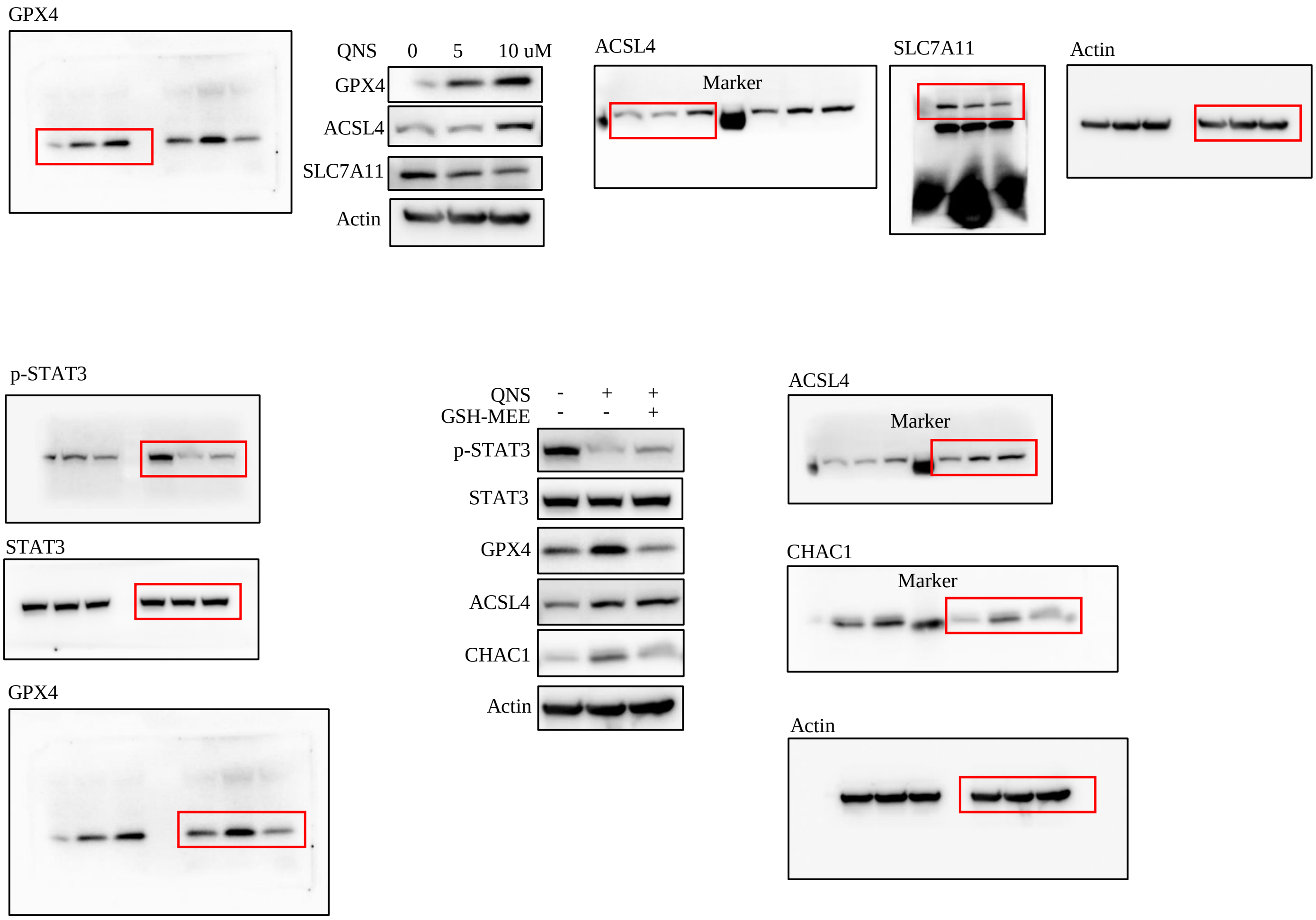

Original uncropped image

Sup Fig 3

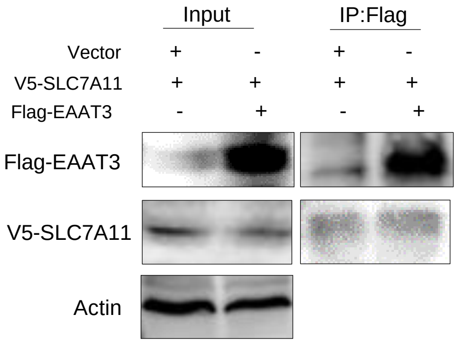

Input

Flag-EAAT3

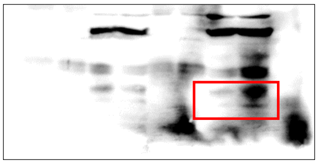

IP:Flag

Flag-EAAT3

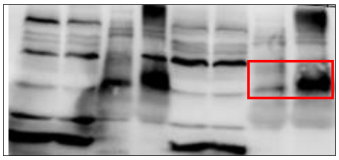

Input

V5-SLC7A11

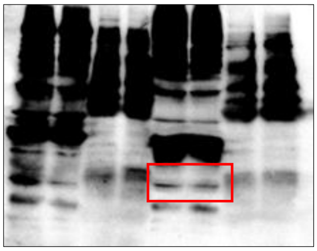

IP:Flag

V5-SLC7A11

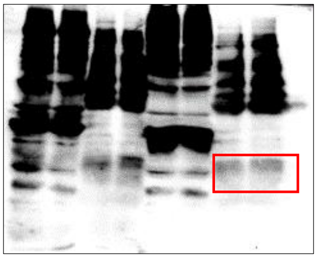

Actin

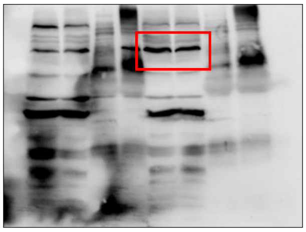

Supplement: Supplementary file 9 — Western blot no-cut Raw data [file 41419_2025_7453_MOESM9_ESM.pdf]
